# A breast tissue-specific epigenetic clock provides accurate chronological age predictions and reveals de-correlation of age and DNA methylation in tumor-adjacent and tumor samples

**DOI:** 10.1080/15592294.2026.2714582

**Published:** 2026-09-11

**Authors:** Leonardo D. Garma, Sonia Pernas, Bartomeu Fullana, Andrea Vethencourt, David Vicente Baz, Teresa García Manrique, Rosario García Campelo, Cristina Reboredo, Josefa Terrasa, Antonia Perello, Ramón Colomer, Desirée Jiménez, Ruth Vera García, Susana de la Cruz Sánchez, Begoña Bermejo, Marta Tapia, Santiago González Santiago, Miguel Quintela-Fandino

**Affiliations:** a Breast Cancer Clinical Research Unit, Centro Nacional de Investigaciones Oncologicas (CNIO), Madrid, Spain; b Medical Oncology Department, Institut Catala d’Oncologia (ICO)-Institut d’Investigació Biomèdica de Bellvitge (IDIBELL), Barcelona, Spain; c Hospital Virgen de la Macarena, Sevilla, Spain; d Medical Oncology Service, University Hospital A Coruña (XXIAC-SERGAS), A Coruña, Spain; e Department of Medical Oncology, Hospital Son Espases, Palma de Mallorca, Spain; f Medical Oncology Division, Hospital Universitario La Princesa, Madrid, Spain; g Department of Medicine, Universidad Autónoma de Madrid (UAM), Madrid, Spain; h Precision Medicine, Universidad Autonoma de Madrid (UAM – Fundación Instituto Roche), Madrid, Spain; i Medical Oncology Department, Hospital Universitario de Fuenlabrada, Fuenlabrada, Spain; j Medical Oncology Department, Hospital Universitario de Navarra, Pamplona, Spain; k Medical Oncology, Hospital Clínico Universitario de Valencia, Biomedical Research Institute INCLIVA, Medicine Department, Universidad de Valenciaencia, Biomedical Research Institute INCLIVA, Valencia, Spain; l Medical Oncology, Hospital San Pedro de Alcántara, Cáceres, Spain

**Keywords:** Breast cancer, epigenetic clocks, aging, breast, DNA methylation, tumor-adjacent

## Abstract

Epigenetic clocks have been widely used to estimate biological age across various tissues, but their accuracy in breast tissue remains suboptimal. Classical models such as Horvath’s and Hannum’s clocks perform poorly in predicting chronological age in breast tissue, underscoring the need for a tissue-specific approach. In this study, we introduce a Breast Tissue-specific Epigenetic Clock (BTEC), developed using DNA methylation data from 553 healthy breast tissue samples across seven different studies. BTEC significantly outperformed existing clocks, demonstrating superior correlation with chronological age (*r* = 0.91) and lower prediction errors (MAE = 3.0 y) without requiring dataset-specific regressions adjustments. Notably, BTEC showed consistent performance across ancestry groups, in contrast to existing clocks which exhibited variable ancestry-related biases. We also found that residual-based epigenetic age acceleration, commonly used to correct for prediction bias, is highly dependent on the choice of reference dataset and does not provide a generalizable correction. BTEC’s chronological age predictions for tumor-adjacent samples showed distortions, with an average deviation of −2.3 y, which was even more pronounced in tumor samples, where the average difference between predicted and chronological age was −14 to −15 y. When analyzed by molecular subtype, the distortion was greater in the more aggressive HER2+ and TNBC tumors compared to HR+ tumors. Overall, our findings indicate that breast tumors do not generally exhibit accelerated epigenetic aging. The probes used by BTEC were associated with known oncogenes such as JAK1, TP73, PDGFRA, CCND1 and STAT5B, as well as genes involved in cancer-related pathways, including Wnt, Hedgehog and ErbB signaling. Importantly, extreme deviations in epigenetic age, as measured by BTEC-derived epigenetic age acceleration (EAA), were associated with patient survival in HR+ breast cancer patients in an exploratory analysis, highlighting the prognostic value of tissue-specific epigenetic aging measures. These findings demonstrate that BTECs not only improve age prediction in breast tissue but also capture biologically meaningful alterations in tumor epigenetic aging with potential clinical implications.

## Introduction

Aging is a major risk factor for numerous diseases [1,2], and amongst the largest risk factors for cancer [3]. As women age, their risk of developing breast cancer increases substantially, with incidence rising sharply until menopause and continuing to climb at a slower rate thereafter [4]. This age-related risk is driven by a combination of factors, including accumulated genetic mutations, prolonged exposure to hormones, and changes in breast epithelium [5].

Epigenetic changes have been found to have a strong relationship with aging [6] and some authors have even suggested that epigenetic modifications are the main hallmark of the aging process [7]. This relationship led to the development of epigenetic clocks or *epiclocks* [8–10] which are mathematical models that predict chronological age based on the methylation state of specific regions in the DNA [11]. The difference or deviation between the predicted (or *biological* or *epigenetic*) age and the actual chronological age (i.e., the prediction error) is commonly referred to as epigenetic age acceleration (EAA) and is often interpreted as a measure of aging rate or biological aging. Epigenetic clocks have been used to link EAA to multiple features and pathologies, from cancer [12,13] to psychiatric disorders [14–16] or socio-economic status [17,18]. However, recent research suggests that epigenetic clocks designed to estimate chronological age can be effectively constructed using blood DNA methylation patterns even at random CpG sites [19,20]. In contrast, clocks aimed at capturing specific biological traits associated with aging would need to be based on measurement of non-stochastic, biologically regulated methylation events [19,20]. Therefore, we hypothesize that accurately measuring biological age in breast tissue and uncovering the molecular changes associated with aging would require the development of a novel, tissue-specific epigenetic clock. An accurate breast-specific biological aging clock could be valuable in several clinical contexts, such as refining risk assessment in screening programs, predicting risk of breast cancer development, contributing to a better understanding of breast aging and hormonal influence, and in the case of malignant transformation, clarify the understanding of epigenetic aging changes from before to after transformation as well as their therapeutic implications.

The Horvath model was developed using multi-tissue data and was originally intended as a multi-tissue predictor [9]. Other popular epigenetic clocks, such as Hannum’s and Levine’s models, were developed using blood DNA methylation data but were attributed multi-tissue capabilities [10,21]. However, the developers of these models observed low performances on breast tissue: Horvath reported a correlation coefficient of 0.73 between the epigenetic age calculated by its model and chronological age in 23 normal breast tissue samples of the training set of his model, and noted that the model is poorly calibrated in breast tissue. Likewise, Hannum’s clock reached a correlation of 0.72 when tested on data from 83 tumor-adjacent breast tissue samples from the TCGA, and the authors suggested that the intercept and slope of the model needed to be re-adjusted for breast. In the case of PhenoAge, the correlation was much lower, reaching only 0.42. Subsequent studies testing multiple clocks reported correlations between 0.35 and 0.78, confirming the limited capability of these models to work with breast tissue data [22–24].

Despite these limitations, these models have been applied to breast-tissue data (regressing them on age to provide a better fit) in order to address several clinical questions of relevance. To date, they have been used to suggest that DNA methylation age is elevated in breast tissue of healthy women [9,25,26] that there is significant epigenetic age acceleration (EAA) in tumor-adjacent compared to normal breast tissue [22,23] and that tumor tissue presents higher epigenetic age [10] than the host. Ren et al. used raw epigenetic age (predicted by Horvath’s model) and found that it was associated with menopausal status, better breast cancer prognosis and with ER/PR-negative, HER2-enriched, and basal-like subtypes [27]. However, given that Horvath’s model shows weak correlation with chronological age in breast tissue (see above), and that epigenetic age in this study covaried strongly with menopausal status and molecular subtype, it remains unclear whether these associations reflect a genuine epigenetic aging process or are instead driven by subtype-specific methylation differences that the clock’s chronological-age correlate happens to capture.

Recently, a deep-learning pan-tissue epigenetic clock found that breast tumors exhibited significantly increased EAA compared to normal breast tissue, with an average acceleration of 4.542 y, on a dataset comprising 100 samples [28]. Also, a high-performance tissue-specific model by Castle et al. reported no difference in EAA between normal and tumor-adjacent samples, and increased EAA in tumor samples [29]. Notably, the authors claimed that late-staged tumors exhibited a non-significant negative EAA [29]. However, Castle et al.‘s model was developed using targeted bisulfite sequencing of a custom set of CpG probes, and is therefore not directly applicable to commonly available array-based methylation data (450k/EPIC).

Here, we evaluated the performance of various pan-tissue epigenetic clocks on a large and diverse DNA methylation dataset from breast tissue of healthy donors, consisting of 553 samples across 7 different studies. After all models produced low-accuracy chronological age predictions, we developed the Breast Tissue-specific Epigenetic Clock (BTEC), which significantly improved chronological age predictions for healthy tissue samples. When applied to tumor and tumor-adjacent samples, BTEC’s predictions were notably distorted, generally showing reduced epigenetic age acceleration (EAA) and contradicting findings from previous models. This distortion was more pronounced in tumor samples than in tumor-adjacent tissues, with the greatest effect observed in triple-negative breast cancer (TNBC) tumors, followed by HER2+ tumors, and the least in hormone receptor-positive (HR+) cases. Despite the overall decrease in EAA in tumors compared to normal breast tissue, the relationship between BTEC’s predictions and cancer survival indicated that TNBC tumors with increased epigenetic ages had significantly lower survival.

## Results

### Breast tissue DNA methylation data

**Figure 1.**
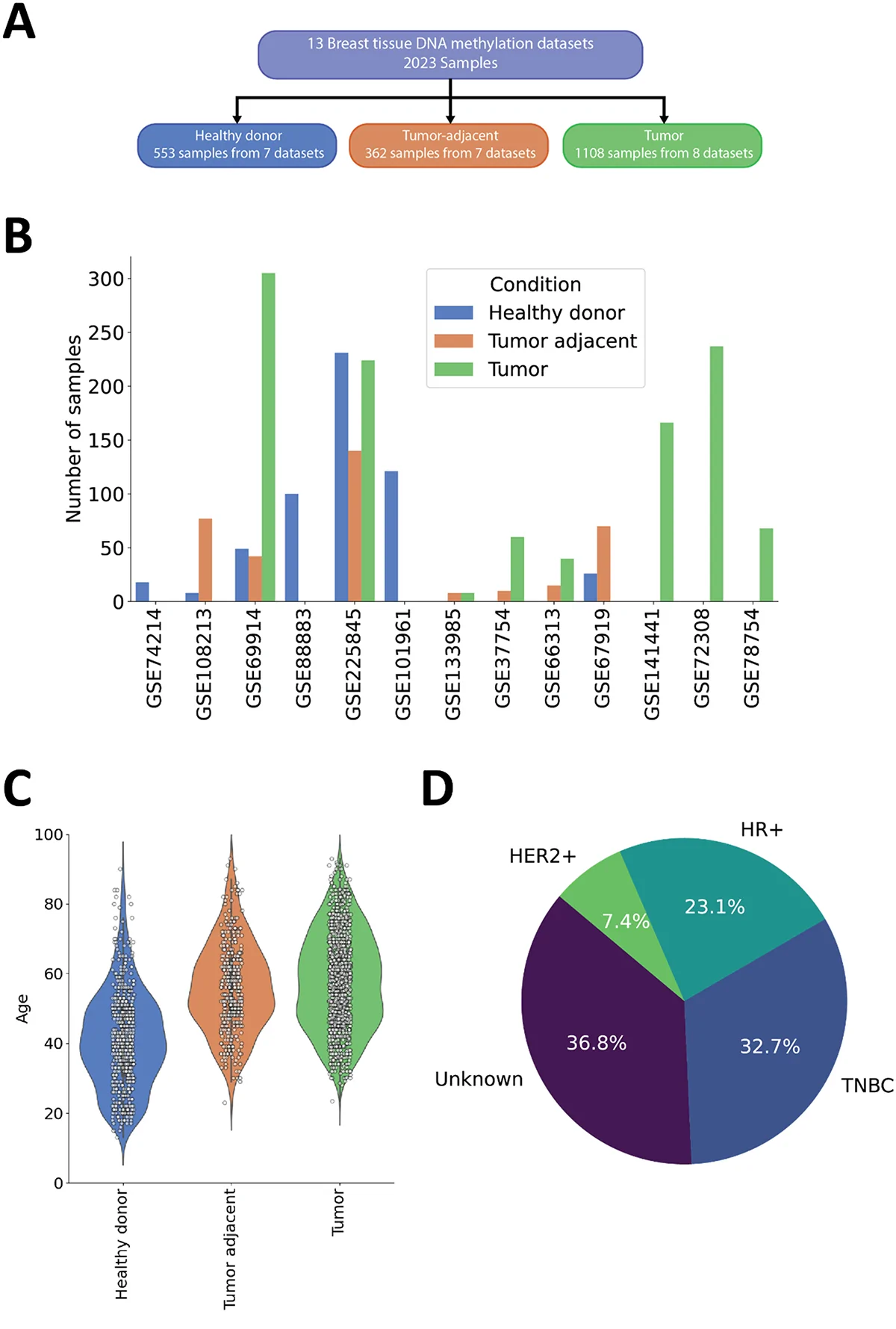
A. Summary of the data collected. B. Number of samples per study and type included in the dataset. C. Distribution of subject ages per sample type. D. Distribution of tumor samples per breast cancer type.

Previous studies examining epigenetic age in breast tissue have relied on relatively small datasets, ranging from 23 to 200 [9,23,28] subjects, limiting the robustness and generalizability of the models. This limitation is evident in the varying correlations between predicted and chronological age reported both in the original studies and in subsequent applications. To improve upon these models, we compiled a larger dataset by integrating breast tissue DNA methylation data from multiple sources, generated using Illumina’s 450k and EPIC DNA methylation arrays. Our dataset includes samples from female donors from 13 different studies, comprising 553 normal (healthy donor) samples, 362 tumor-adjacent samples, and 1108 tumoral samples from female subjects. The healthy donor samples were later split into training and validation sets to develop new epigenetic clocks (see below) (Figure 1(A,B)).

The age of the healthy sample donors ranged from 13 to 90 y, while the tumor and tumor-adjacent samples were obtained from subjects with ages between 24 and 93 y (Figure 1(B)). Out of the 1108 tumor samples, 700 were labeled with a specific molecular subtype: 256 hormone-receptor positive (HR+), 82 *HER2*-positive (HER2+) and 362 triple-negative breast cancer (TNBC) samples (Figure 1(D)).

Ancestry metadata (self-reported race) was available in five datasets (GSE225845, GSE101961, GSE37754, GSE67919, GSE108213; Supplementary Table 1) for a subset of samples across the healthy, tumor-adjacent, and tumor cohorts. Among healthy samples, 208 were labeled as White (White of European-American), 174 as Black (Black or African-American), and 4 as Other (Asian, Hispanic or Other). In the tumor-adjacent cohort, 156 samples were labeled as White, 153 as Black, and 16 as Other. Among tumor samples, 159 were labeled as Black, 123 as White, and 2 as Other.

The estimated tumor purity of the tumor samples had a median value of 0.548, with an inter-quartile range (IQR) of 0.173, similar to previous studies [30,31]. The purity values on the tumors were significantly higher (*P* = 3.193e-181) than on the tumor adjacent (median = 0.276, IQR = 0.163) and the healthy donor samples (median = 0.284, IQR = 0.17). We did not observe a significant difference (*P* = 0.359) between the purity distributions of the tumor adjacent and the healthy donor samples (Supplementary Figure S1). Detailed information about the data sources and the characteristics of the corresponding cohorts are detailed in Supplementary Table 1.

### Performance of multi-organ epiclocks

We used the combined dataset of 553 samples from healthy donors to evaluate the accuracy of chronological age predictions from four epigenetic clocks: Hannum [10] Horvath [9] PhenoAge [21] and AltumAge [28]. Horvath’s model and AltumAge were trained using multi-tissue data, whereas PhenoAge and Hannum’s model were trained using blood DNA methylation data but were attributed multi-tissue capabilities. We therefore refer to all these models as ‘pan-tissue’ epigenetic clocks. Each of these epigenetic clocks predicted the age of the donors based on breast tissue DNA methylation data (‘pan-to-breast’ prediction) pre-processed using two normalization strategies (BMIQ-normalization via Minfi [32] and wateRmelon [33] or beta values calculation and pre-processing via SeSAMe [34] see Methods). For comparison, we used two baseline models: a naïve model that always predicted the average age of the donors (41.01 y) and a random model which made random predictions within the entire age range of the dataset (between 13 and 90 y).

**Figure 2.**
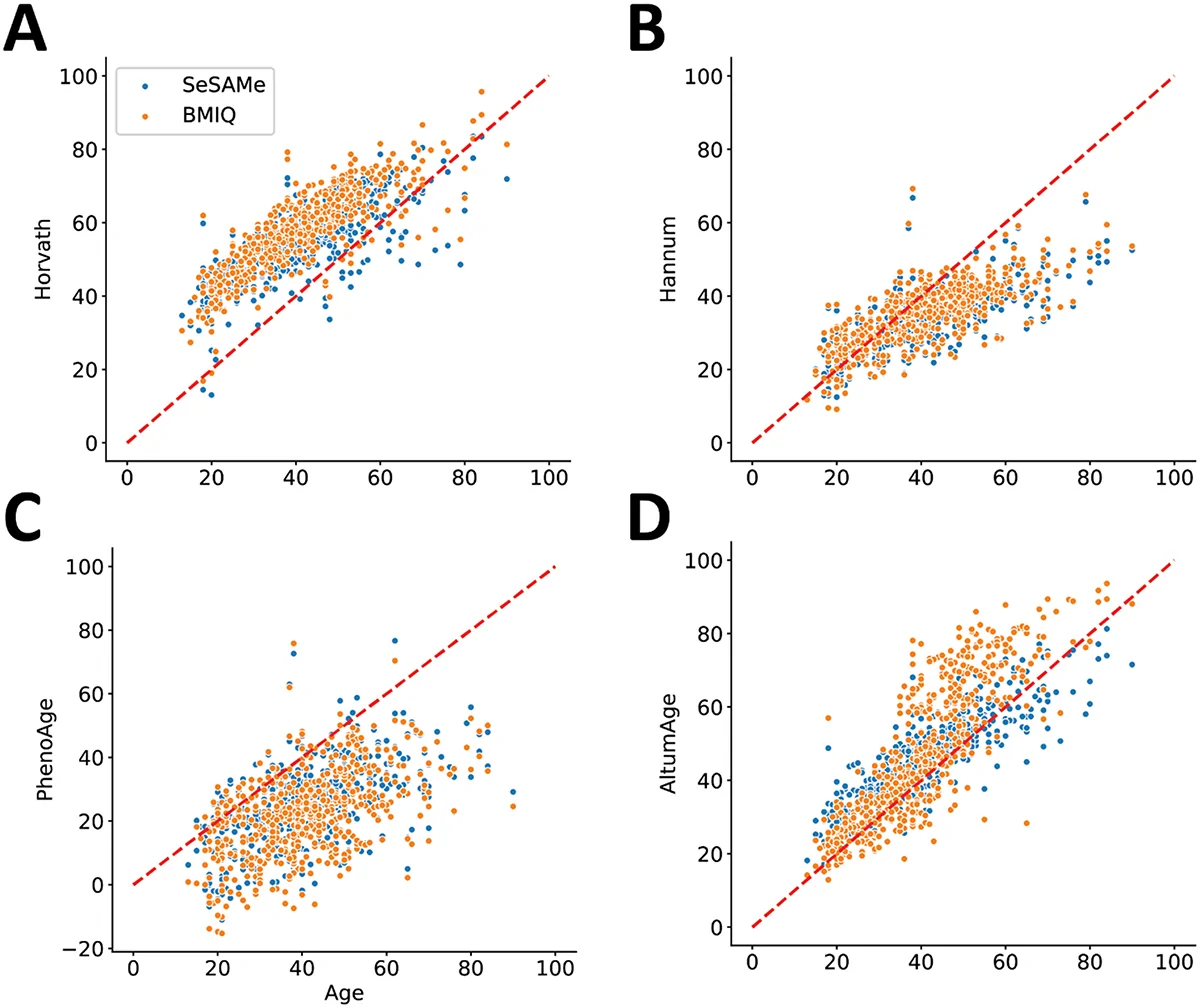
A–D. Chronological age predictions for the breast tissue samples from healthy donors produced by the four models tested: Horvath’s 2013 model, Hannum’s clock, PhenoAge and Altum age. Each dot indicates the actual and predicted age values of one sample, colored by the normalization strategy used. The identity line is represented in red.

The results showed that the four epigenetic clocks produced predictions that were correlated with chronological age (*r* values ranging from 0.522 to 0.873; Figure 2(A-D), Table 1). However, all four models exhibited considerable root-mean‑squared error (RMSE) values, ranging from 8.496 to 22.682 y (Table 1). AltumAge performed the best, with the lowest prediction error (RMSE and median absolute error [MAE]) and highest correlation, followed by Hannum’s model. Notably, the performance of both models was lower when the BMIQ normalization was used. Both Horvath’s model and PhenoAge showed larger errors than the naïve model, both in terms of RMSE and MAE, indicating their relatively low performance. All models outperformed the random predictor.

**Table 1.** Root-mean‑squared error (RMSE), median absolute error (MAE) and Pearsons’ correlation coefficient (*r*) for each model on the healthy donor samples (*N* = 553).

Model	Norm	RMSE (y)	MAE (y)	r
AltumAge	BMIQ	12.917	7.081	0.845
AltumAge	SeSAMe	8.496	6.309	0.873
PhenoAge	BMIQ	22.682	18.084	0.522
PhenoAge	SeSAMe	20.539	15.718	0.540
Hannum	BMIQ	11.246	7.046	0.737
Hannum	SeSAMe	11.946	7.528	0.746
Horvath	BMIQ	18.452	17.658	0.828
Horvath	SeSAMe	16.270	15.342	0.771
Naïve	normal	13.927	9.010	N/A
Random	normal	27.995	19.368	0.063

To identify possible demographic biases, we compared prediction errors by ancestry. We found that AltumAge (SeSAMe) and Horvath’s model (SeSAMe and BMIQ) had significant differences in performance between ancestry groups, with increased error on samples from Black subjects relative to White subjects (FDR < 0.02, *n* = 8 tests; *N* = 174 Black, *N* = 208 White). In contrast, Hannum’s model (SeSAMe and BMIQ) showed a significant (FDR < 0.02) but opposite bias, with increased MAE and RMSE on predictions for White subjects (Supplementary Table S2). These divergent, model-specific biases raise concerns about the ancestry generalizability of existing epigenetic clocks.

Given their limited raw performance, some epigenetic clocks compute EAA as the residuals of a linear regression between predicted and chronological age, fitted on a reference (typically healthy) cohort, rather than as the raw difference between predicted and chronological age. Because this approach depends on the choice of reference dataset, we first tested whether the parameters of this linear fit are stable across different references. For all four clocks tested (AltumAge, PhenoAge, Hannum’s, Horvath’s), both the dataset main effect and the Predicted Age: Dataset interaction term were statistically significant (Type II ANOVA on the fitted linear models; FDR < 0.05 in all cases except the slope term for PhenoAge with SeSAMe normalization), indicating that both the intercept and slope of the regression between chronological age and predicted age differ significantly depending on which dataset is used as reference (Supplementary Tables S3 and S4, Supplementary Figure S2). We then compared prediction error (RMSE, MAE) obtained using residual-based EAA against raw EAA across these reference datasets (Supplementary Figure S3). For AltumAge, Hannum’s, and Horvath’s models, residual-based correction reduced error relative to raw EAA when certain reference datasets were used, but increased error above the raw baseline when other reference datasets were used. This inconsistency indicates that the effect of residualization on prediction accuracy is not a fixed property of a given clock, but is instead driven by the arbitrary choice of reference dataset.

### A breast-tissue-specific epigenetic clock outperforms pan-tissue models

To address the limitations of pan-tissue models, we developed tissue-specific Breast Tissue-specific Epigenetic Clocks (BTECs). For training these models, we utilized 553 healthy donor samples from seven studies.

**Figure 3.**
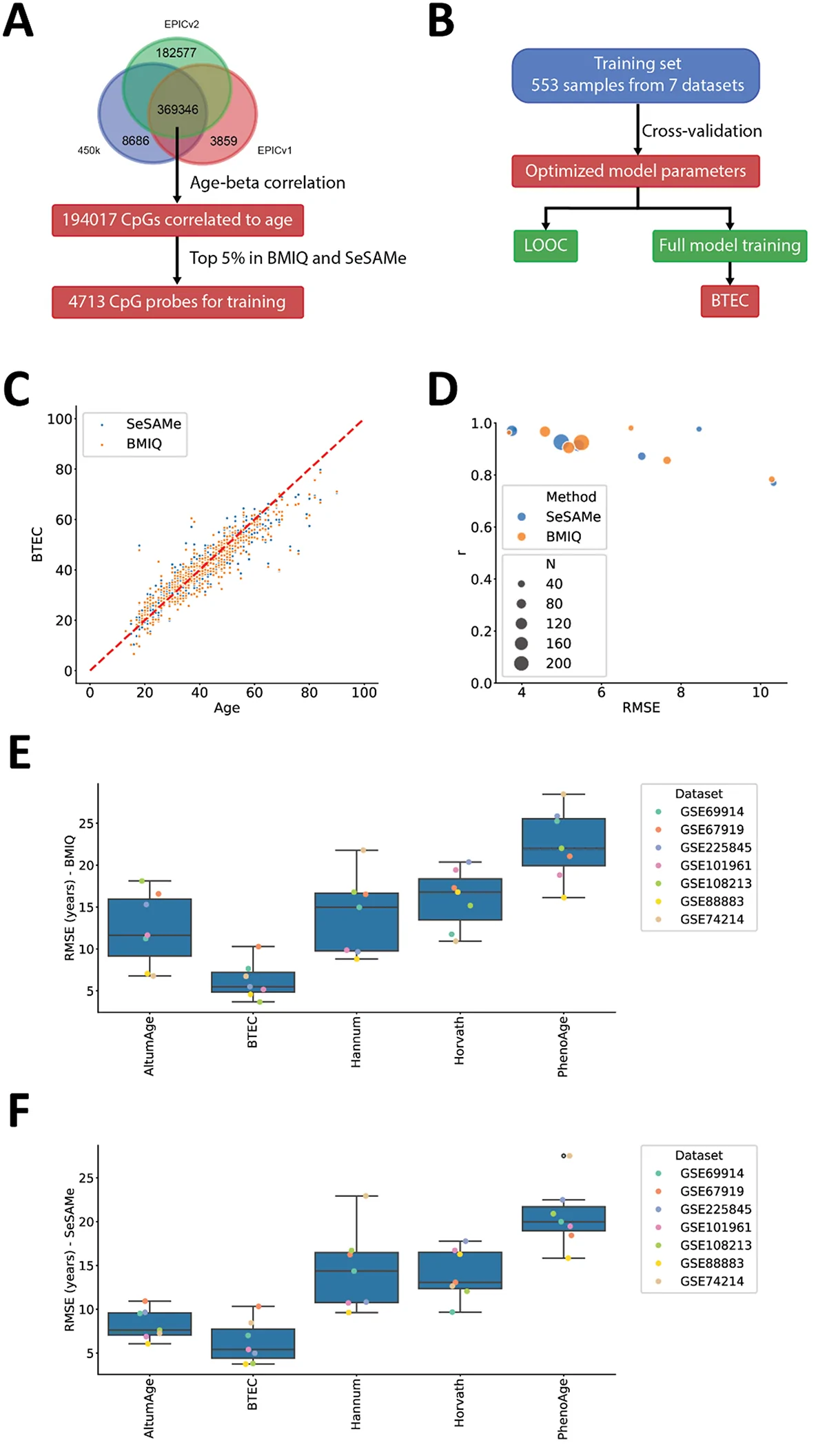
A. Schematic view of the CpG probe selection process. The Venn diagram shows the number of probes shared by the different DNA methylation platforms. The shared probed were tested for linear or quadratic correlation with chronological age and then the top 5% were selected for training the models. B. Schematic view of the strategy used to develop and validate the BTEC models. C. Predictions obtained through the LOCO scheme. BTEC models were trained on *N* − 1 cohorts and then made predictions on the remaining cohort. The plot shows the chronological ages of the subjects and the epigenetic age predicted by the BTECs under this scheme. D. Summary metrics (Pearson’s *r* and RMSE) of the results of the LOCO strategy per dataset, colored by normalization method. The size of the dots indicates the number of samples in each dataset. E. Distribution of the predictions RMSE on the BMIQ-normalized data per dataset. The boxes represent the quartiles of the distribution while the whiskers extend to the largest and the smallest datapoint within 1.5 times the inter-quartile range (Q3–Q1) above Q3 or below Q1 respectively. F. Same as E on the SeSAMe-normalized data.

We employed linear and quadratic terms in constructing the models, using elastic net regression. To select the appropriate CpG probes, we focused on those conserved across the 450k, EPICv1, and EPICv2 microarrays that showed significant (FDR < 0.05) linear or quadratic correlations with chronological age in the training set under either BMIQ or SeSAMe normalizations. From the resulting 370,706 linear and quadratic features from 194,017 CpG probes, we selected the top 5% in terms of absolute Pearson correlation with chronological age under both normalizations (|*r*| > 0.321, *N* = 13,176 for BMIQ and |*r*| > 0.28, *N* = 13,806 for SeSAMe). We used the 4713 probes corresponding to the linear and quadratic features in the top 5% correlations under both normalizations to train the model using elastic net (Figure 3(A); Supplementary Table S5).

We set the L1 ratio to 0.5 and optimized the alpha value to 0.0077 through 5-fold cross-validation on the training set (see Methods) for the BMIQ- and SeSAMe-normalized data. With these optimized parameters, we applied a Leave-One-Cohort-Out (LOCO) approach, where models were trained on 6 out of 7 datasets (*N* − 1) and used to predict chronological age on the excluded cohort (Figure 3(B)).

This approach resulted in a strong correlation (*r* = 0.918 and 0.912 for the SeSAMe and BMIQ‑based models, respectively) between the predicted and chronological ages, and a MAE below 3.2 y (Figure 3(C), Table 2). The performance varied by dataset but remained overall above that of existing models (Figure 3(D-F), Supplementary Table S6).

**Table 2.** Results from each model on the healthy donor samples (*N* = 553). BTEC models trained and tested using the LOCO strategy.

Model	Norm	RMSE (y)	MAE (y)	r
BTEC	BMIQ	5,839	3,188	0,912
BTEC	SeSAMe	5,590	2,947	0,918
AltumAge	BMIQ	12,917	7,081	0,845
AltumAge	SeSAMe	8,496	6,309	0,873
PhenoAge	BMIQ	22,682	18,084	0,522
PhenoAge	SeSAMe	20,539	15,718	0,540
Hannum	BMIQ	11,246	7,046	0,737
Hannum	SeSAMe	11,946	7,528	0,746
Horvath	BMIQ	18,452	17,658	0,828
Horvath	SeSAMe	16,270	15,342	0,771

We further examined whether BTEC’s predictions were affected by ancestry. Comparing the EAAs between Black and White subjects, we found no significant differences in performance (*P* = 0.108 and *P* = 0.08 for the BMIQ and SeSAMe-normalized models, respectively; *N* = 174 Black, *N* = 208 White), indicating that BTEC’s accuracy is consistent across these ancestry groups and does not suffer from the demographic biases observed in other models.

Obesity is a well-established risk factor for postmenopausal, hormone receptor-positive breast cancer, with meta-analytic estimates showing an approximately 40% increase in risk among obese women [35]. Given this established link, we tested whether obesity status similarly biased epigenetic age predictions in the healthy samples. Comparing samples from obese (BMI ≥ 30, *N* = 95) and non-obese subjects (*N* = 176), neither BTEC nor any of the other models tested (AltumAge, Horvath’s, Hannum’s, PhenoAge) showed significant differences in prediction error (*P* > 0.05 and FDR > 0.2 in all cases, *n* = 10 tests), suggesting that obesity status does not substantially bias epigenetic age predictions in any of the models tested (Supplementary Table S7).

After the initial assessment through the LOCO approach, we re-trained the BTEC models on all the healthy samples using the same L1 and alpha hyperparameters. On the BMIQ-normalized data, the resulting BTEC included 917 linear and 1354 quadratic terms derived from 1631 CpG probes, whereas the model trained using SeSAMe-normalized data consisted of 913 linear and 1353 quadratic terms derived from 1617 CpG probes (Supplementary Table S8).

### Epigenetic clocks performance in tumor-adjacent samples

We then evaluated the performance of the models on tumor-adjacent (TA) samples. First, we considered the 392 TA samples from our integrated dataset, followed by 44 TA samples from The Cancer Genome Atlas (TCGA) [36] (excluding 53 samples present in AltumAge’s training set, see Methods).

**Figure 4.**
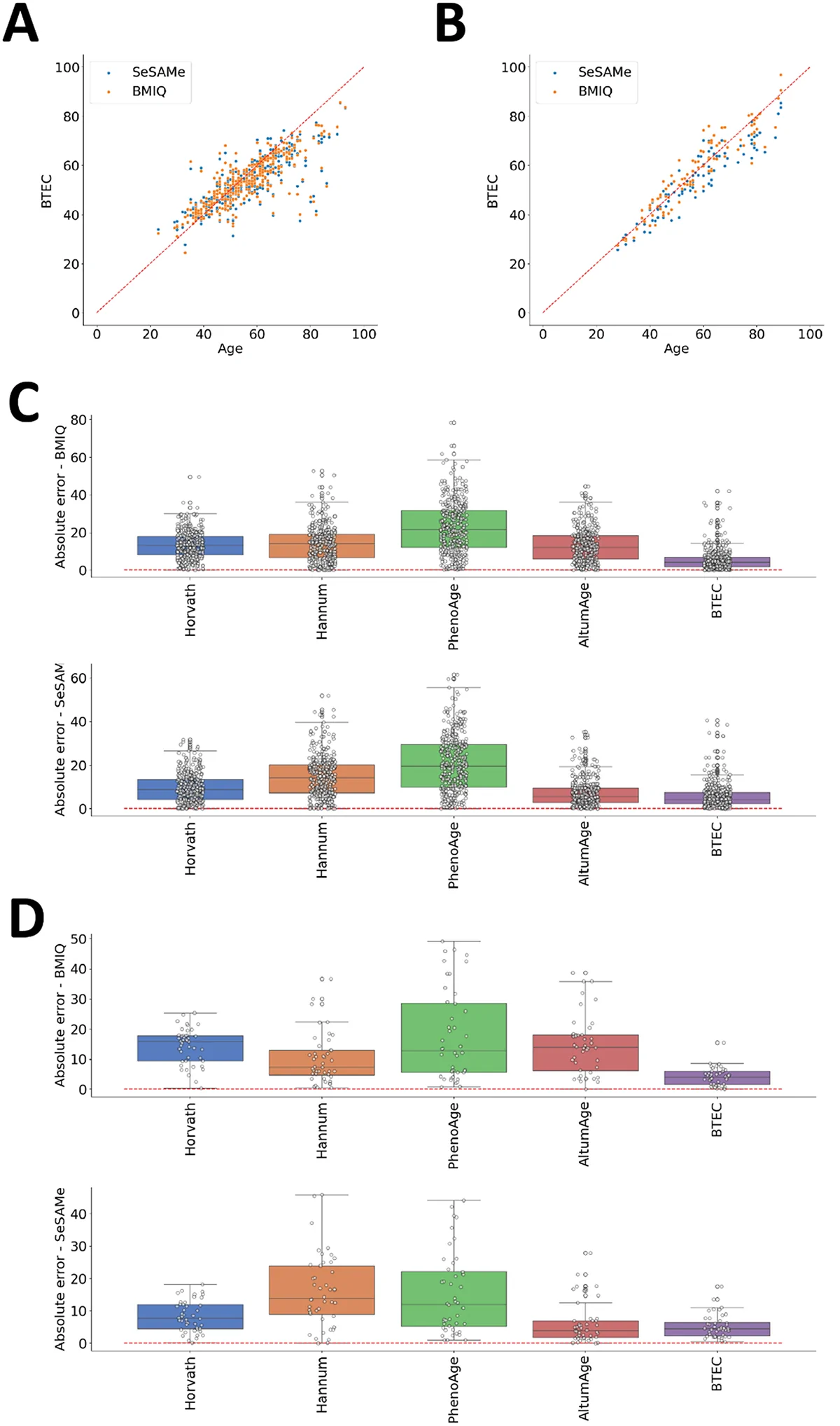
A. BTECs’ results on the tumor adjacent samples from the integrated dataset (*N* = 362, colored by normalization). B. BTECs’ results on data from the TCGA samples (*N* = 44), colored by normalization. C. Distribution of absolute prediction error on the tumor adjacent samples using BMIQ- (top) and SeSAMe-normalized data. The boxes represent the quartiles of the distribution. The whiskers extend to the largest and the smallest datapoint within 1.5 times the inter-quartile range (Q3-Q1) above Q3 or below Q1 respectively. D. Distribution of absolute prediction error on the TCGA samples using the two different normalization strategies. The box‑plot elements have the same interpretation as in C.

The performance of the BTEC models decreased on this data; however, they still produced lower RMSE values than the rest of the models (Figure 4, Tables 3 and 4). The increase in age prediction errors could be due to real epigenetic alterations between TA and healthy tissue samples, implying that TA tissue departs biologically from healthy donor samples. On the integrated dataset’s TA samples, which are external to all the models, the BTECs exhibited significantly lower error distributions (FDR < 0.05 in all cases: range 4.14 × 10^−76^ -PhenoAge- to 2.27 × 10^−42^ -AltumAge- on BMIQ-normalized data; 9.56 × 10^−67^ -PhenoAge- to 1.92 × 10^−4^ -AltumAge- on SeSAMe-normalized data; *n* = 8 tests) compared to all other models.

**Table 3.** Results on tissue adjacent samples (*N* = 362).

Model	Norm	RMSE (y)	MAE (y)	r
BTEC	BMIQ	8,010	4,310	0,820
BTEC	Sesame	8,229	4,343	0,808
AltumAge	BMIQ	15,673	12,234	0,700
AltumAge	SeSAMe	9,449	5,709	0,725
PhenoAge	BMIQ	26,677	21,553	0,276
PhenoAge	SeSAMe	24,612	19,557	0,257
Hannum	BMIQ	16,849	14,040	0,491
Hannum	SeSAMe	17,390	14,409	0,477
Horvath	BMIQ	15,239	13,181	0,719
Horvath	SeSAMe	11,592	8,788	0,713

**Table 4.** Results on TCGA samples (*N* = 44).

Model	Norm	RMSE (y)	MAE ()	r
BTEC	BMIQ	5,122	4,041	0,951
BTEC	Sesame	6,346	4,517	0,941
AltumAge	BMIQ	17,446	14,038	0,852
AltumAge	SeSAMe	8,799	4,028	0,847
PhenoAge	BMIQ	23,157	12,796	0,478
PhenoAge	SeSAMe	19,510	12,055	0,528
Hannum	BMIQ	12,941	7,379	0,809
Hannum	SeSAMe	19,546	13,965	0,753
Horvath	BMIQ	15,520	15,842	0,911
Horvath	SeSAMe	9,628	7,792	0,882

The TA set comprised 369 fresh frozen and 23 FFPE-preserved samples. Because FFPE preservation can introduce DNA degradation and bisulfite conversion artifacts that may confound methylation-based measurements, we compared the distributions of EAA estimates for each model between the two preservation methods to assess whether preservation method introduced a detectable bias. We compared the distributions of EAA estimates for each model between the two preservation methods and found that only PhenoAge clocks resulted in significant differences (FDR < 0.05, *n* = 10 tests; Supplementary Table S9). As our BTEC models were not among those affected, we did not apply any correction for preservation method in subsequent analyses.

We also examined whether any model’s performance in the TA cohort was affected by ancestry. Comparing prediction EAA distributions between Black and White subjects (*N* = 126 Black, *N* = 153 White), Hannum’s model (both normalizations) and PhenoAge (BMIQ) exhibited significant differences by ancestry (FDR < 0.05, *n* = 10 tests) while AltumAge (SeSAMe) and Horvath’s model (BMIQ) presented weakly significant biases (*P* < 0.05, FDR < 0.2, *n* = 10 tests; Supplementary Table S10).

Comparing obese (BMI ≥ 30) and non-obese subjects, only Hannum’s model (both normalizations) presented a weak bias (*P* < 0.05, FDR < 0.2, *n* = 10 tests), with increased RMSE in non-obese subjects (Supplementary Table S11). BTEC models (SeSAMe and BMIQ-based) showed no significant differences by ancestry or obesity status in the TA cohort.

On the TCGA samples, BTEC outperformed all other models in terms of both RMSE and *r*. This difference in RMSE was statistically significant for every model comparison (FDR < 0.05, *n* = 8 tests), with the exception of AltumAge on SeSAMe-normalized data, where the difference did not reach significance (*P* = 0.064, FDR = 0.064; Figure 4(D), Table 4).

### Epigenetic age alterations in tumor-adjacent and tumor samples

EAA is typically calculated as the difference between the age predicted by an epigenetic clock and the chronological age. However, due to the poor performance of pan-tissue epigenetic clocks on breast tissue DNA methylation data, several studies have instead defined EAA as the residuals from a linear regression of the predicted epigenetic age on chronological age [10,22–24]. As shown above, this approach is an ad-hoc solution: the regression is computed on a per-study or per-dataset basis, and its fitted parameters vary significantly depending on which dataset is used as reference, making residual-based EAA neither a principled correction nor comparable across studies. Since the BTECs produced accurate age predictions with low prediction errors for healthy tissue, we chose to compute EAA directly as the difference between predicted and chronological ages.

**Figure 5.**
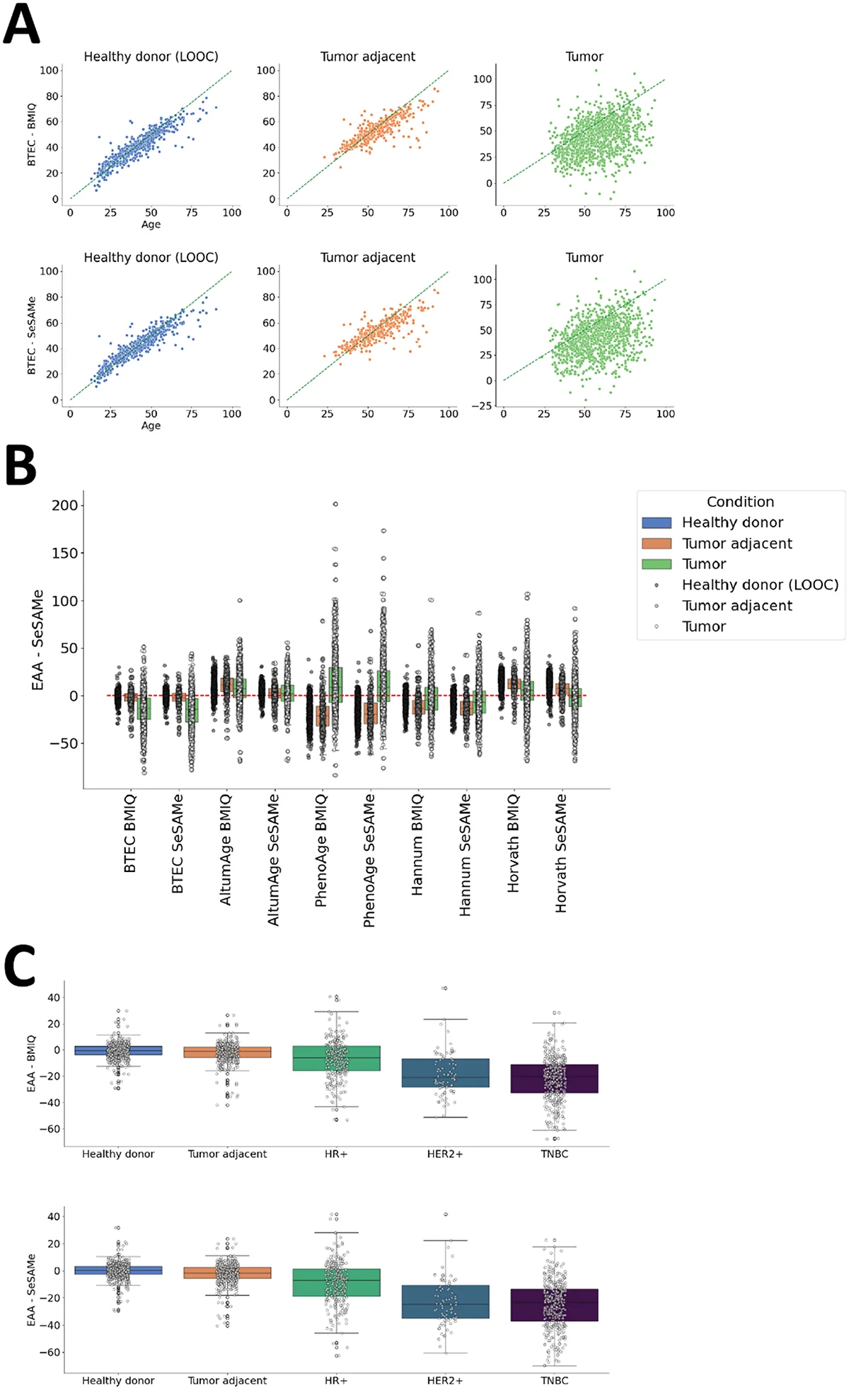
A. Epigenetic age predictions from BTEC on breast tissue samples from healthy donors (left), tumor adjacent samples (middle) and breast tumors (right). The first row shows the results on the BMIQ-normalized data, and the second those obtained from the SeSAMe-normalized values. B. EAA distributions by sample type, epigenetic clock and normalization strategy. The boxes represent the quartiles of the distribution while the whiskers extend to the largest and the smallest datapoint within 1.5 times the inter-quartile range above Q3 or below Q1 respectively. C. Distribution of epigenetic age accelerations predicted by both BTECs’s broken down by tumor type. The box‑plot elements have the same interpretation as in B.

The BTECs’ predictions for cancer patient samples were generally lower than the chronological ages (Figure 5(A)). Both tumor-adjacent and tumor samples had significantly lower EAAs compared to normal samples (*P* = 0.007 and *P* = 5.16 × 10^−69^ for the TA and Tumor samples respectively on the BMIQ-normalized data; *P* = 8.34 × 10^−34^ and *P* = 1.07 × 10^−85^ in the case of SeSAMe-normalized data). The average EAAs on the BMIQ-normalized data were −2.358 y (range: −42.018 to 26.533) for tumor-adjacent samples and −13.938 y (range: −81.064 to 51.419) for tumor samples, whereas for healthy samples, the average EAA (estimated using the LOCO approach) was −0.978 y. A similar trend emerged when we used the SeSAMe-normalized data (Table 5).

**Table 5.** Characteristics of the EAA distributions on the different sample types from the integrated dataset, excluding healthy samples used for the BTECs’ training.

Model	Norm	Condition	N	Mean EAA (y)	Std (y)	Min EAA (y)	Max EAA (y)
BTEC	BMIQ	Healthy donor (LOOC)	553	−0,978	5,762	−29,639	29,821
BTEC	BMIQ	Tumor	1108	−13,938	17,356	−81,064	51,419
BTEC	BMIQ	Tumor adjacent	392	−2,358	7,665	−42,018	26,533
BTEC	Sesame	Healthy donor (LOOC)	553	−0,017	5,595	−29,431	31,642
BTEC	Sesame	Tumor	1108	−15,432	18,308	−77,803	43,873
BTEC	Sesame	Tumor adjacent	392	−2,329	7,903	−40,652	23,478
AltumAge	BMIQ	Healthy donor (LOOC)	553	7,338	10,641	−36,680	40,131
AltumAge	BMIQ	Tumor	1108	8,003	16,257	−68,480	100,160
AltumAge	BMIQ	Tumor adjacent	392	10,963	11,215	−44,631	40,823
AltumAge	SeSAMe	Healthy donor (LOOC)	553	5,031	6,852	−22,280	30,772
AltumAge	SeSAMe	Tumor	1108	2,478	13,881	−67,704	55,754
AltumAge	SeSAMe	Tumor adjacent	392	2,209	9,199	−35,426	33,955
PhenoAge	BMIQ	Tumor	1108	13,208	29,712	−83,568	201,416
PhenoAge	BMIQ	Tumor adjacent	392	−20,205	17,442	−66,056	78,546
PhenoAge	BMIQ	Healthy donor (LOOC)	553	−18,366	13,323	−65,407	37,813
PhenoAge	SeSAMe	Healthy donor (LOOC)	553	−16,031	12,852	−60,843	34,620
PhenoAge	SeSAMe	Tumor	1108	12,423	27,228	−76,096	173,355
PhenoAge	SeSAMe	Tumor adjacent	392	−18,089	16,711	−61,499	68,044
Hannum	BMIQ	Healthy donor (LOOC)	553	−5,861	9,607	−37,496	31,295
Hannum	BMIQ	Tumor	1108	−2,610	19,881	−63,691	100,724
Hannum	BMIQ	Tumor adjacent	392	−11,761	12,080	−50,519	52,697
Hannum	SeSAMe	Healthy donor (LOOC)	553	−7,063	9,644	−38,799	28,780
Hannum	SeSAMe	Tumor	1108	−5,819	18,695	−61,881	86,565
Hannum	SeSAMe	Tumor adjacent	392	−12,599	12,002	−51,927	42,464
Horvath	BMIQ	Healthy donor (LOOC)	553	16,714	7,823	−23,556	43,973
Horvath	BMIQ	Tumor	1108	5,601	19,145	−68,346	106,766
Horvath	BMIQ	Tumor adjacent	392	12,032	9,363	−27,882	49,591
Horvath	SeSAMe	Tumor	1108	−1,462	17,436	−68,124	91,861
Horvath	SeSAMe	Healthy donor (LOOC)	553	13,627	8,897	−30,397	41,806
Horvath	SeSAMe	Tumor adjacent	392	6,768	9,423	−30,761	31,643

The pan-tissue models largely reproduced this phenomenon: only PhenoAge (both normalizations) and Hannum’s model (BMIQ) assigned significantly lower EAAs to the healthy samples compared to the tumor samples (FDR < 0.05; one-sided Wilcoxon test, Benjamini–Hochberg FDR-corrected, *n* = 10 tests; Figure 5(B), Table 5, Supplementary Figure 4). Thus, contrary to the claims of some studies [10,26,28] the BTECs results indicate that breast tumors have a negative distortion of epigenetic age. Moreover, the other models do not reveal a consistent pattern of accelerated epigenetic aging in either tumor adjacent or tumor samples relative to healthy samples in this large dataset (Figure 5(B)).

When tumor samples were categorized by molecular subtype, BTEC predictions assigned significantly lower EAA (*P* = 2.735 × 10^−9^ and *P* = 5.391 × 10^−11^ on the BMIQ- and the SeSAMe-normalized data, respectively; two-sided Wilcoxon test) to HER2+ samples compared to HR+ samples, with TNBC cases exhibiting the largest negative EAA (Figure 5(C,D), Table 6).

**Table 6.** Characteristics of the BTECs’ EAA distributions on the tumor samples, by tumor molecular type.

Model	Norm	Molecular_type	N	Mean EAA (y)	Std (y)	Min EAA (y)	Max EAA (y)
BTEC	BMIQ	Healthy donor	553	−0,978	5,762	−29,639	29,821
BTEC	BMIQ	Tumor adjacent	392	−2,358	7,665	−42,018	26,533
BTEC	BMIQ	HR+	256	−7,018	14,714	−53,252	40,401
BTEC	BMIQ	HER2+	82	−18,262	16,679	−51,078	47,019
BTEC	BMIQ	TNBC	362	−22,215	16,630	−67,693	28,388
BTEC	Sesame	Healthy donor	553	−0,017	5,595	−29,431	31,642
BTEC	Sesame	Tumor adjacent	392	−2,329	7,903	−40,652	23,478
BTEC	Sesame	HR+	256	−9,022	15,941	−62,425	41,615
BTEC	Sesame	HER2+	82	−22,757	17,761	−60,520	41,759
BTEC	Sesame	TNBC	362	−25,310	17,159	−69,928	22,479

To explore this observation further, we compared the EAA with the PAM50 classification of tumors on two datasets with available annotations (GSE72308) or RNA-seq data (GSE225845). On the GSE225845 we observed that the EAA of the Basal samples was significantly lower than that of the Luminal A (*P* = 0.018 and *P* = 0.012 using BMIQ and SeSAMe normalization respectively; two-sided Wilcoxon test; FDR < 0.05 in both cases, Benjamini–Hochberg FDR-corrected, *n* = 8 tests). On the GSE72308 dataset, the EAA of the basal samples was significantly lower than that of either the Luminal A or B samples (*P* = 2.735 × 10^−11^ to *P* = 7.131 × 10^−8^ and *P* = 4.885 × 10^−12^ to *P* = 2.171 × 10^−8^ for Luminal A and B using BMIQ and SeSAMe-normalized data; FDR < 0.05 in all cases, Benjamini–Hochberg FDR-corrected, *n* = 8 tests). Unfortunately, the datasets with PAM50 subtype classifications did not contain enough HER2+ samples to extend the comparison to these subtypes (Supplementary Figure S5).

To determine whether the observed tumor-associated EAA differences could be explained by cell-type composition rather than a genuine epigenetic signal, we estimated epithelial, fibroblast, and immune cell fractions for each tumor sample using EpiDISH [37] and tested their association with BTEC-derived EAA. Epithelial fraction showed a weak or non-significant correlation with EAA (BMIQ: *r* = −0.040, *P* = 0.185; SeSAMe: *r* = −0.113, *P* < 0.001), while fibroblast fraction was moderately positively correlated (BMIQ: *r* = 0.274; SeSAMe: *r* = 0.322; *P* < 10^−19^ for both) and immune cell fraction moderately negatively correlated with EAA (BMIQ: *r* = −0.135; SeSAMe: *r* = −0.098; *P* < 0.01 for both; Supplementary Table 12). A linear model of EAA as a function of Epi and Fib fractions explained only a small proportion of the overall variance (*R*^2^ = 0.075 for BMIQ, *R*^2^ = 0.107 for SeSAMe), with Fib as the only consistently significant predictor (Supplementary Table 13). When molecular subtype was added to this model, the subtype effect on EAA remained highly significant in both normalizations (Type II ANOVA; BMIQ: *F* = 31.49, *P* = 1.57 × 10^−19^; SeSAMe: *F* = 43.78, *P* = 9.84 × 10^−27^), and its contribution to the total sum of squares was several-fold larger than that of either cell-type covariate (Supplementary Table S14). These results indicate that cell-type composition does not account for the tumor subtype-associated differences in BTEC-derived EAA.

To further characterize the tumor-associated epigenetic age findings, we examined BTEC-derived EAA in relation to the clinicopathological variables most consistently annotated across the public datasets used in this study: histologic grade and tumor stage. Among HR+ tumors, EAA did not differ significantly between Grade 1 (*n* = 51) and Grade 3 (*n* = 61) tumors (*P* = 0.241 and *P* = 0.275 on BMIQ- and SeSAMe-normalized data, respectively). Grade distribution was highly skewed in TNBC (127 Grade 3, 5 Grade 2, 1 Grade 1) and HER2+ tumors (47 Grade 3, 8 Grade 2), precluding a statistically meaningful comparison in these subtypes. Regarding tumor stage, EAA did not differ significantly across Stages I, II, and III in either HR+ (ANOVA, *P* = 0.088 and *P* = 0.105 for BMIQ- and SeSAMe-normalized data, respectively) or TNBC tumors (ANOVA, *P* = 0.266 and *P* = 0.408 for BMIQ- and SeSAMe-normalized data, respectively). The set of clinicopathological covariates available per dataset is provided in Supplementary Table S1.

We additionally tested whether treatment history (radiation, chemotherapy, hormone therapy) was associated with EAA within each molecular subtype, using the treatment annotations on datasets GSE141441 and GSE7754. The comparisons revealed that the BTECs assigned significantly lower EAAs to TNBC tumors treated with chemotherapy or radiation. The number of subjects with treatment annotations in the HR+ cohort (*N* = 0 radiation, *N* = 18 hormone therapy, *N* = 10 chemotherapy) prevented us from considering the analysis for this tumor type robust or clinically meaningful (Supplementary Table S15).

### Tumor epigenetic age is not determined by the replication rate

The idea that methylation age measures the number of somatic cell replications was introduced as ‘a plausible hypothesis’ by Horvath [9] and then refuted for his model. To explore a possible explanation for our findings of decelerated biological aging in breast tumors, we investigated whether the predicted epigenetic age of tumors predicted by BTEC correlates with the number of cell replications. The expression of Ki-67, a well-known biomarker of cell proliferation [38], is widely used in oncology to assess tumor aggressiveness [39]. In breast cancer specifically, Ki-67 levels are used to classify hormone receptor-positive (HR+) tumors into the Luminal A and Luminal B subtypes [40].

Here, we used the Ki-67 annotations available in three of the datasets we collected (GSE69914 [41], GSE141441 [42] and GSE72308 [43]) to explore whether this biomarker was related to the chronological age of the donors, their epigenetic age predicted by BTEC, or their EAA. Ki67 data were not reported individually in the study from Gao et al. [41] instead, patients were grouped into two categories based on their Ki-67 status: low (patients with Ki-67 below 14%) and high (patients with Ki-67 at or above 14%). This classification, based on a 14% cut-off, comes from a previous large study in which luminal-type breast cancer showed distinct clinical courses and endocrine sensitivity depending on this threshold [44]. To enable direct comparisons, we applied the same classification criteria (Ki-67 below or above 14%) to the other datasets.

**Figure 6.**
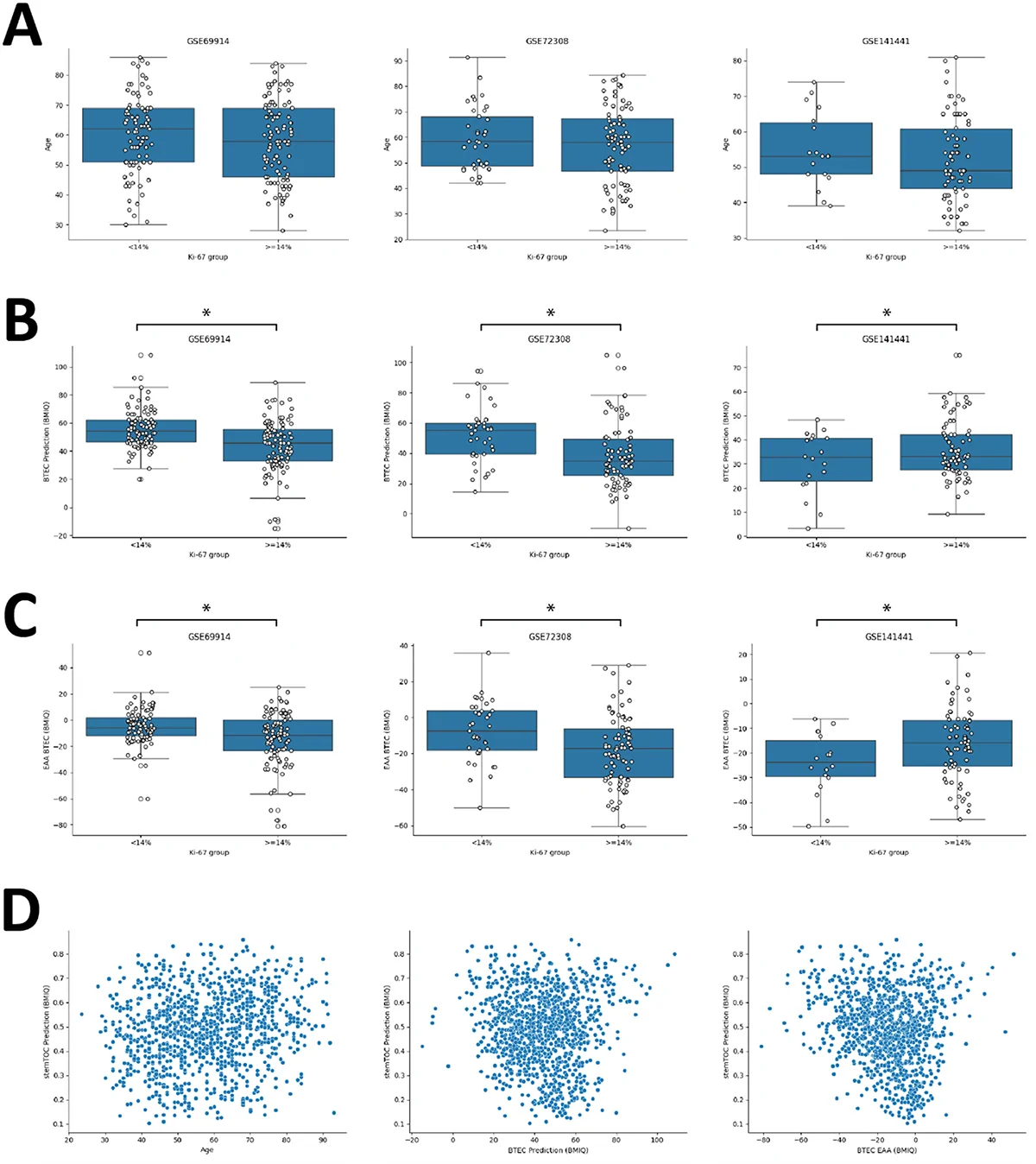
A. Distribution of ages by Ki-67 group on three different datasets (BMIQ-normalized data). B. Distribution of predicted epigenetic age by Ki-67 group on the same datasets. C. Distribution of EAA by Ki-67 group on the same datasets. D. stemToc’s predicted mitotic age plotted against chronological age (left), BTEC’s epigenetic age (middle) and BTEC’s EAA on all the tumor samples of the integrated dataset (*N* = 1108, BMIQ normalization).

We observed that the distribution of chronological ages did not significantly differ between the Ki-67 groups (*P* > 0.05, two-sided Wilcoxon test) on any of the datasets. Patients with higher Ki-67 values exhibited significantly lower epigenetic ages (*P* = 7.668 × 10^−6^ and *P* = 9.771 × 10^−6^ on BMIQ- and SeSAMe-normalized data respectively on GSE69914; *P* = 3.757 × 10^−4^ and *P* = 5.422 × 10^−4^ in the case of GSE72308; two-sided Wilcoxon test; Benjamini–Hochberg FDR < 0.05 in all cases, *n* = 6 tests) and EAAs (*P* = 9.069 × 10^−4^ and *P* = 1.207 × 10^−3^ on BMIQ- and SeSAMe-normalized data respectively on GSE69914; *P* = 3.319 ×10^−3^ and *P* = 2.602 ×10^−3^ in the case of GSE72308; two-sided Wilcoxon test; Benjamini–Hochberg FDR < 0.05 in all cases, *n* = 6 tests) in two of the datasets. Contrary to the assumption that accelerated biological aging is a result of repeated replication cycles, these findings would suggest the opposite: tumors with lower epigenetic ages and EAAs may retain the highest replicative potential. However, in the dataset from Fackler et al. [42] we observed contradictory results, as the samples with high Ki-67 values exhibited significantly higher EAA values (Figure 6(A–C); Supplementary Figure 6A,B).

The lack of consistency and contradictory results in these studies suggest that there is no clear relationship between the epigenetic age or the EAA determined by BTEC and the Ki-67 values. This implies that the tumors’ epigenetic age predicted by BTEC is not directly determined by the number of replications the tumor cells have undergone.

We next applied a mitotic clock, a tool designed to estimate the cumulative number of cell divisions a cell population has undergone, to investigate the relationships between chronological age, epigenetic age, EAA, and the estimated number of cell replications in tumor samples. Mitotic age predictions were obtained for all tumor samples in the dataset using stemTOC [45]. No significant correlations were observed between stemTOC predictions and chronological age, the BTEC predictions, or EAA (Figure 6(D); Supplementary Figure 6C). Likewise, in tumor-adjacent and healthy samples used for BTEC validation, no clear associations between stemTOC and these variables were detected (Supplementary Figure 7A, B). In addition, we observed similar results when we tested Horvath’s model and AltumAge (Supplementary Figure 7C). These findings suggest that mitotic age, as estimated by stemTOC, is largely decoupled from both chronological and epigenetic ages.

### BTECs’ probes methylation states decouple from age in tumor‑adjacent and tumor samples

To gain insight into the differences observed in the epigenetic age predictions for healthy tissue, tumor-adjacent, and tumor samples, we examined the methylation state of the probes used by the BTECs across the different sample types.

**Figure 7.**
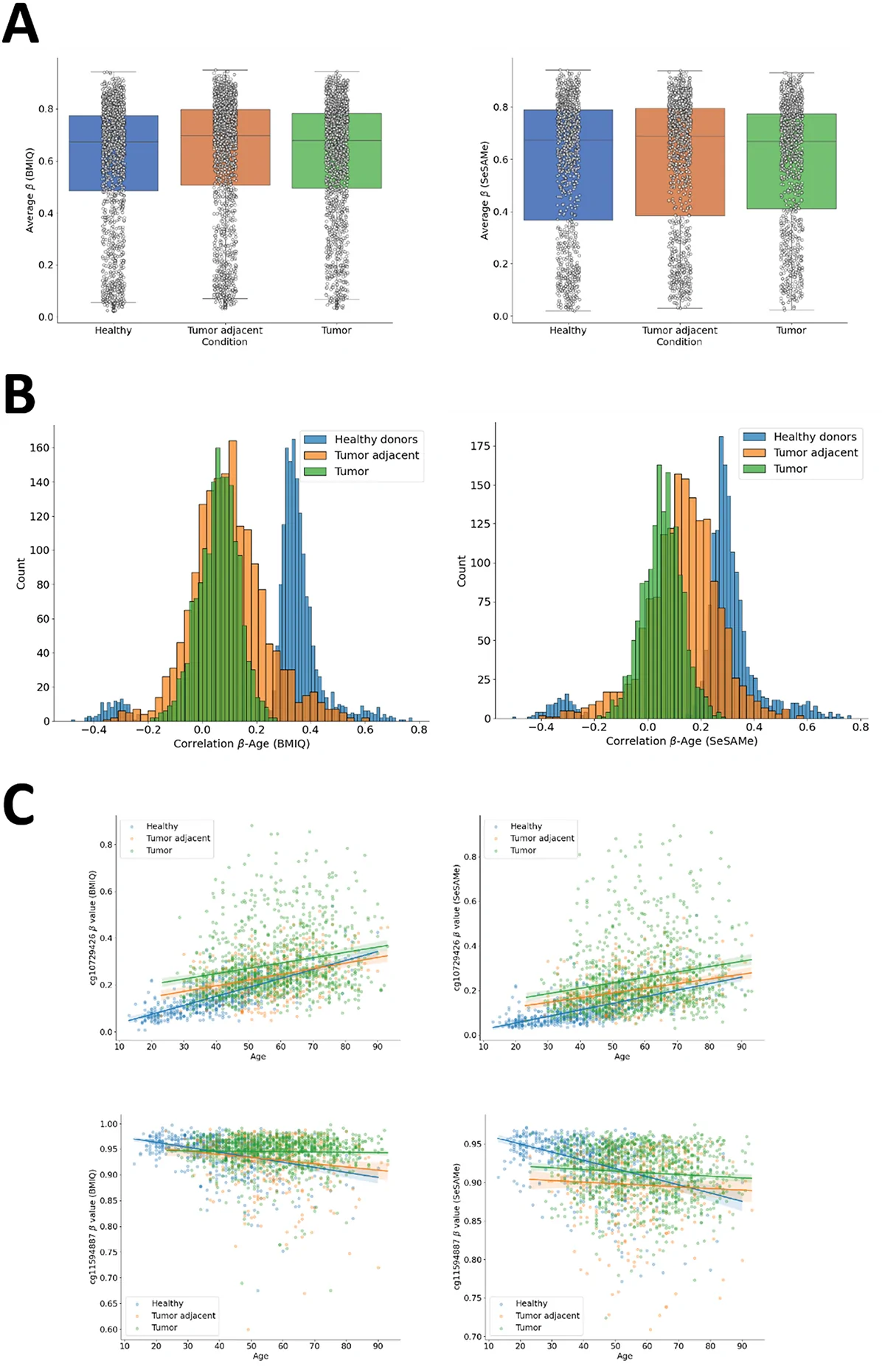
A. Distribution of average methylation beta values on the probes used by the BMIQ-based (left) and the SeSAMe-based (right) BTECs on the three sample types. The boxes represent the quartiles of the distribution while the whiskers extend to the largest and the smallest datapoint within 1.5 times the inter-quartile range above Q3 or below Q1 respectively. B. Distribution of correlations between the beta values of the BTECs probes used by BTEC and chronological age, colored by sample type. The left panel shows the values obtained from the BMIQ-normalized data and the right panel shows the results from the SeSAMe-normalized data. C. Examples of probes with divergent methylation-age correlations in the different sample types on both BTECs. Each dot represents a sample, indicating the beta value of the probe and the subject’s age. The overlaid lines are the corresponding linear regressions.

Overall, the average methylation levels on the BTECs probes were higher in the tumor-adjacent and tumor samples compared to the healthy tissue samples (Figure 7(A)).

Upon examining individual probes, we found that 1512 and 1149 probes in the tumor-adjacent samples in the BMIQ-normalized and the SeSAMe-normalized data, respectively, had significantly different distributions of methylation beta values (FDR < 0.05) compared to the samples from the healthy donors. Likewise, 1480 and 1475 probes in the tumor samples had significant differences in the BMIQ-normalized data (Supplementary Table 16). and the SeSAMe-normalized.

Moving beyond the absolute methylation values, we observed that the relationship between the methylation states of BTEC’s probes and chronological age was very different across the three sample groups. The feature pre-selection and the regularization applied during BTEC’s training resulted in probes which had average absolute correlations (|*r*|) with age of 0.359 and 0.321 (using BMIQ- and SeSAMe-normalized data respectively) in the healthy donor samples. However, these correlations were considerably weaker in the tumor-adjacent (means |*r*|=0.130 and |*r*|=0.158) and tumor samples (mean |*r*|=0.078 and |*r*|=0.074; Figure 7(B), Supplementary Table 17). In these altered tissues, the models’ probes lost the correlations with age that they exhibited in the healthy donor samples (Figure 7(C)). This loss of correlation likely explains the distortions observed in the predictions for tumor-adjacent and tumor samples.

### BTECs probe-associated genes highlight cancer pathways and model-specific functions

**Figure 8.**
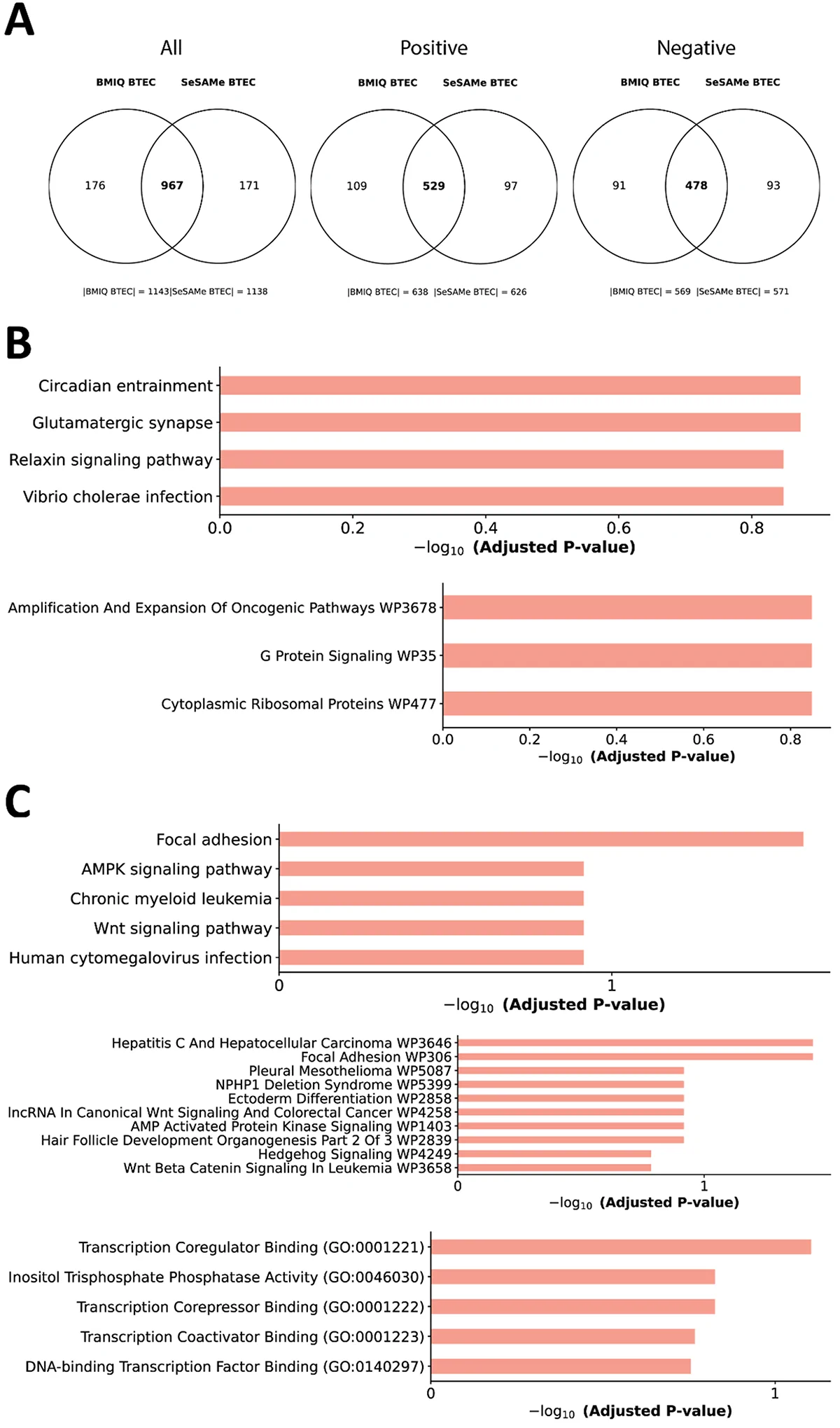
A. Genes mapped to the BTECs’ probes shared by the two models. The left diagram shows the number of genes associated to all the probes; the middle one considers only probes with positive coefficients; and the right one, only probes with negative coefficients. B. KEGG (top) and WikiPathways (bottom) terms enriched in the set of genes associated to positive probes shared by both BTECs. C. KEGG (top), WikiPathways (middle) and GO molecular function (bottom) terms enriched in the set of genes associated to negative probes shared by both BTECs.

We then explored the genes related to the BTECs’ probes and their associated molecular biological functions. We examined the genes mapped to the probes positively and negatively correlated with age on both models and observed that the gene sets of both BTECs had a large (>73%) overlap (Figure 8(A); Supplementary Table S18). According to the GenAge database [46], the common sets included only 4 genes (*SHC1*, *TP53*, *HTT*, and *ADCY5*) with direct evidence linking it to aging in mammals. Considering the genes associated to the individual models (Supplementary Table 18) added only four other genes with strong links to aging: *EPS8*, GHR, and *MTOR*. This indicates that neither model relies predominantly on genes with known associations to aging.

**Figure 9.**
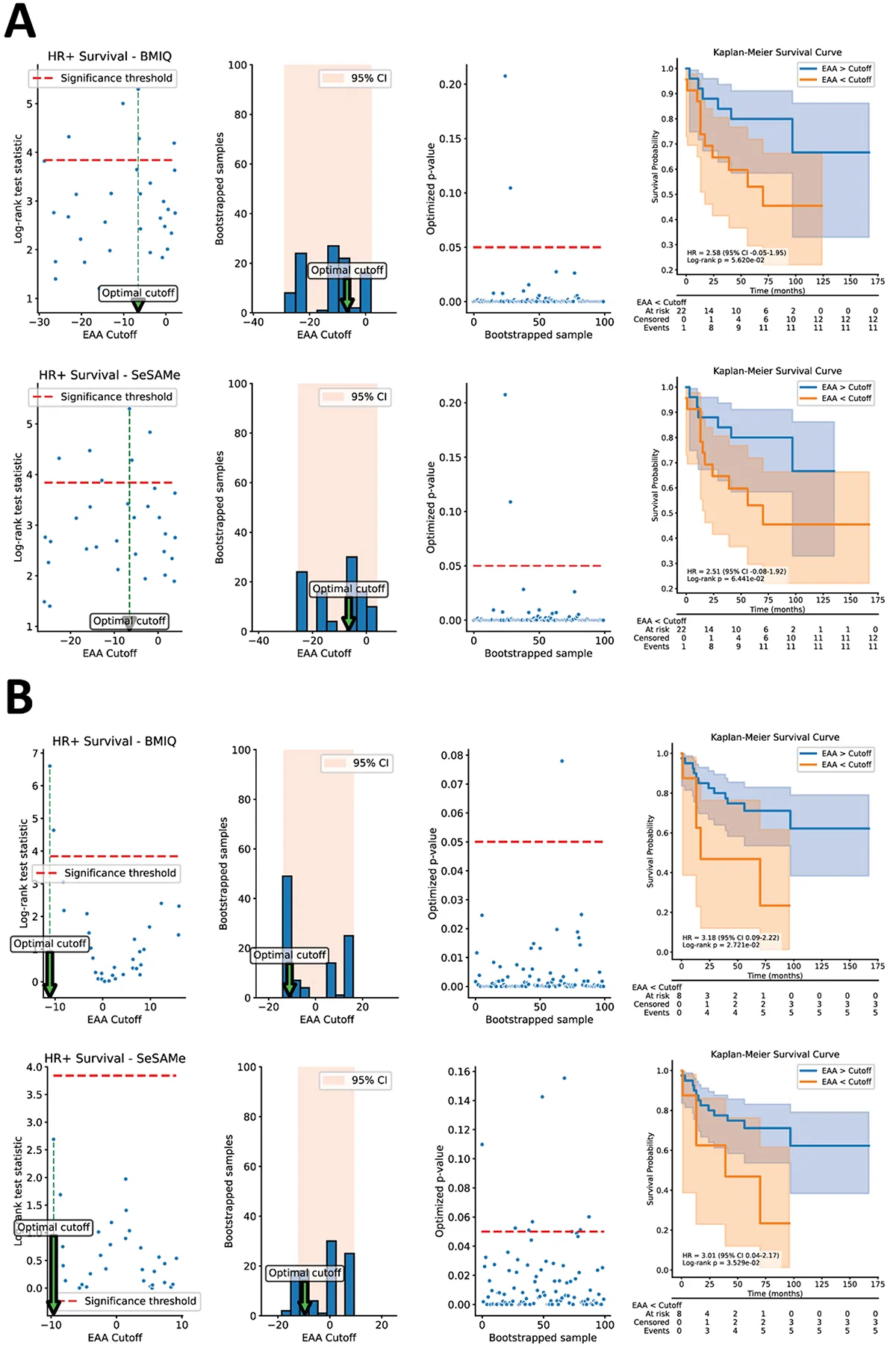
A. Top – log-rank test statistic value obtained using different BMIQ-based BTEC’s EAA cutoff values to split patients from the HR+ survival dataset (GSE37754) into two groups and comparing their survival times (left). Bootstrapping results on the same subjects, showing the distribution of optimized EAA cutoff values and the corresponding *P*-values across the 100 bootstrapped samples (middle two panels). Kaplan–Meier survival curves for patients with EAA above or below −6.58 y (right). The green arrows indicate the value of the optimal cutoff found in the original data (−6.58 y); the dashed red line marks the significance thresholds for the log-rank test statistic and the *P*-value (3.85 and 0.05 respectively); the confidence interval of the optimal EAA cutoff found across the 100 bootstrapped samples (−28.796 to 1.936 y) is highlighted in pink. Bottom – Same as the top panels, presenting results obtained from the BTEC on the SeSAMe-normalized data. B. Results obtained on the GSE37754 using AltumAge with BMIQ- and SeSAMe-normalized data.

Instead, the over-representation analysis of the genes mapped to positive probes used by both models revealed enrichment in protein translation (cytoplasmic ribosomal proteins), G protein-mediated signaling, oncogenic signaling amplification, and neuronal/synaptic signaling. The genes related to negative probes were enriched in cell adhesion (focal adhesion), developmental signaling pathways (especially Wnt, Hedgehog, and ErbB), AMPK signaling, transcriptional regulation (transcription factor/coactivator/corepressor binding), and multiple cancer-related terms (Figure 9(B, C)).

Most notably, the genes linked to the negative terms on both models included the oncogenes *JAK1*, *TP73*, *PDGFRA*, *CCND1*, *CCND2*, *WWTR1 (TAZ)* and *STAT5B*.

Although both models had gene sets enriched in similar terms (G protein signaling, and the closely related Circadian entrainment) associated to their positive probes, the genes associated to the negative probes presented enrichment in divergent terms. For instance, while the set from the BMIQ-based model was enriched in focal adhesion, the one from the SeSAMe-based BTEC was enriched in Wnt signaling terms (Figure 8(B,C) and Supplementary Figure S8; Supplementary Table S19).

### Age-associated gene expression signatures linked to BTECs’ probes

We examined the RNA expression data from the dataset GSE102088, generated in the breast tissue DNA methylation study from Song et al. [47] (GSE101961), to identify genes involved in BTEC’s predictions that also exhibited expression changes correlated with the subjects’ age. We identified a total of 140 genes that showed a significant and non-weak (FDR < 0.05, |*r*|>0.25) correlation between expression and age (Supplementary Table S20).

Among these, 5 genes were associated with probes that had positive coefficients in both BTECs (Supplementary Table S21): PTGFR, RANBP17, COBL, ELF5, and MVP. This set included ELF5, a transcription factor with an established role in mammary luminal cell differentiation and breast cancer subtype determination [48,49], and MVP, a marker of multidrug resistance previously linked to poor chemotherapy response and estrogen receptor interaction in breast cancer [50]. The remaining three (PTGFR, RANBP17, COBL) lack breast-cancer-specific functional data in the literature to date.

Another 3 genes matched those associated with probes that had negative coefficients in BTEC: VGLL4 (a transcriptional cofactor in the Hippo signaling pathway which is a known tumor-suppresor in breast cancer), COBL and TXNL4B (Supplementary Table S21). Notably, COBL was associated with probes carrying opposite-sign coefficients in the BTEC models: a positive coefficient at cg21355793 and a negative coefficient at cg03551807. This is not unusual, as individual CpGs within or near the same gene can be regulated independently and show divergent methylation trajectories with age depending on their specific genomic context (e.g., promoter, gene body, or CpG island/shore location); as such, the sign of a probe’s coefficient reflects the local behavior of that specific CpG rather than a single, gene-level direction of effect [51,52].

Overall, the overlap between the genes associated with age in BTEC and those identified in the expression data was small, as expected due to the complex relationship between DNA methylation and gene expression [53–55].

However, both data sources highlighted two genes with established roles in breast cancer biology: ELF5, a transcription factor that governs mammary luminal cell differentiation and is a key determinant of breast cancer molecular subtype, and VGLL4, a transcriptional cofactor of the Hippo signaling pathway with a known tumor-suppressor role in breast cancer.

### Transcriptomic signatures of tumors stratified by epigenetic age acceleration

To better understand the differences between tumors with accelerated (EAA > 0) or decelerated (EAA < 0) epigenetic ages as predicted by BTEC, we analyzed the transcriptomic data from Terunuma et al.‘s study [56] (GSE37751 and GSE37754). The differential expression analysis on the tumors stratified using the predictions of the BMIQ-based BTEC revealed that tumors with EAA < 0 over-expressed 130 genes (FDR < 0.2, log(fold change) > 1), including regulators of cell cycle and mitosis (*CDC20*, *CCNA2, PLK1, CDK1*) and immune‑function regulators (*IL2RA*). In contrast, tumors with EAA > 0 were enriched in 193 genes (FDR < 0.2, log(fold change) > 1), including PI3k-Akt signaling genes (*NTRK2*, IGF1R, AREG, FGF1) and genes with prognostic value (*SCUBE2*, *PGR*) (Supplementary Table S22).

When tumors were split following the predictions of the SeSAMe-based BTEC, we found 197 genes overexpressed (FDR < 0.2, log(fold change) > 1) in the samples with EAA < 0.

Out of these, 123 matched with the results from the BMIQ-based BTEC, including the cell cycle regulators *KIF4A*, *IQGAP3*, and *HIST2H3A*, and the immune function genes *FCGR3A*, *IL18*, *CXCL10*, *GBP5*, *TFEC*. Tumors with EAA > 0 overexpressed (FDR < 0.2, log(fold change) > 1) 300 genes, out of which 169 (56.3%) matched with the results obtained using the BMIQ-based model. The matching genes included *NTRK2*, *AREG*, *FGF1*, and *PGR* (Supplementary Table 23).

### BTEC’s EAA magnitude shows an exploratory association with long-term prognosis

To investigate whether the tumors’ epigenetic aging rates assigned by BTEC are linked to clinical outcomes, we analyzed data from three datasets with clinical annotations: GSE141441 [42] which includes relapse-free times for 164 TNBC patients, GSE78754 [57] which provides survival times for 63 TNBC patients, and GSE37754 [56] which is annotated with survival times for 70 patients with different tumor subtypes.

Our findings showed that when modeled as a continuous covariate in the Cox proportional hazards model, the EAA was not significantly associated with clinical outcomes. Given this null result, we conducted a post hoc, exploratory analysis to characterize whether a non-linear association between EAA and risk might exist. When patients were stratified by different EAA cutoffs, the resulting log-rank test statistic exhibited a non-monotonic pattern (Figure 9). We emphasize that this cutoff-scanning approach is hypothesis-generating and was not prespecified; it does not correct for multiple comparisons across the range of cutoffs tested, and any single cutoff identified this way should not be interpreted as a validated clinical threshold.

Among HR+ subjects, stratification using a BMIQ-based EAA threshold of −6.58 y resulted in two groups with significant survival differences (*P* = 0.0214, log-rank test; Figure 9(A)). As an internal consistency check, we computed the *P*-value of the optimal cutoff in 100 bootstrap samples drawn from the same dataset (see Methods). A cutoff yielding a *P*-value below 0.05 was found in every bootstrapped sample, and the distribution of optimal EAA cutoffs remained narrow, with a median of −6.575 y (95% c.i. −28.796 to 1.936 y; Figure 9(A)). Similarly, the SeSAMe-based EAAs presented an optimal cutoff at −6.544 y that separated subjects into two groups with significant survival differences and could be validated via bootstrapping. Using the AltumAge with the BMIQ-normalized data, we also found a suitable cutoff at the edge of the distribution −11.10 y, 95% c.i. −13.46 to 16.01 y; however, the SeSAMe-normalized data did not yield positive results (Figure 9(B)). In contrast with the BTECs, classifying HR+ patients by EAA using the Horvath model did not yield any relevant cutoffs showing comparable internal consistency (Supplementary Figure 9).

In the case of the TNBC patients, none of the models provided suitable EAA cutoffs to separate subjects with significant differences in either survival or relapse risk.

These results highlight that threshold effects are not monotonic, suggesting that distinct biological mechanisms may underlie risk at different EAA levels, and that simply segregating patients into positive versus negative EAA groups may be overly simplistic. The BMIQ- and SeSAMe-based models capture different signals because they rely on distinct sets of CpG probes linked to different regulatory regions and genes (Figure 8, Supplementary Figure S8). As a result, each model may be optimal in specific tumor contexts but not in others. Given the exploratory nature of this analysis, these survival findings should be regarded as hypothesis-generating rather than confirmatory. Importantly, the BTEC-based models identify exploratory prognostic patterns in HR+ cancers consistently across data-normalization strategies, underscoring their added clinical value, pending validation in independent, adequately powered cohorts.

## Discussion

Epigenetic clocks have emerged as valuable tools to estimate biological age based on DNA methylation patterns [11,58]. However, existing epigenetic clocks have demonstrated poor performance in breast tissue: early models reported correlations below 0.75 between predicted and chronological ages and large chronological age prediction errors [9,10]; and the accuracy of state-of-the-art epiclocks reported a wide range of variation across datasets, with reported *r* values between −0.703 and 0.858 [28]. One possible explanation for this limitation is the scarcity of breast tissue-specific DNA methylation data, which has likely hindered the development of models that accurately capture the epigenetic aging process in this tissue. Yet, despite their shortcomings, classical models with claimed pan-tissue capabilities continue to be widely used in studies involving both normal and pathological breast samples [22–25].

To address the limitations of existing models, we compiled a large and diverse dataset from 13 different studies, representing the most comprehensive collection of breast tissue DNA methylation data to date. Using this dataset, we tested four pan-tissue epigenetic clocks. Our results confirmed that these models exhibited poor predictive performance and systematic errors, with blood-based models performing worse than a naïve approach that predicts a constant age. This highlights the limitations of applying generalized epigenetic clocks to breast tissue and underscores the necessity of tissue-specific models. To overcome these issues, we developed BTECs, breast tissue-specific epigenetic clocks, which significantly outperformed all tested pan-tissue models. We trained our models using data processed through two different normalization strategies (BMIQ and the default of QCDPB processing of SeSAMe – see Methods) to provide results comparable with studies using either approach.

Our finding that the linear fit underlying residual-based EAA varies significantly across reference datasets, and that residualization can either improve or worsen prediction error for the same clock depending on which reference is chosen, argues against treating residual-based EAA as a generalizable correction. Because no universal reference cohort exists for this purpose, any reported benefit of residualization is contingent on a dataset-specific choice that cannot be justified on principled grounds. We therefore consider residual-based EAA, as commonly implemented, to be an ad hoc adjustment rather than a robust solution to age-dependent prediction bias, and caution against its use without an explicit, prespecified, and broadly validated reference standard.

These ancestry-associated differences in prediction error further underscore the limited generalizability of existing pan-tissue clocks. Notably, the direction of the bias was not consistent across models: AltumAge and Horvath’s clock overestimated age (or showed elevated error) in Black subjects relative to White subjects, whereas Hannum’s clock showed the opposite pattern. This inconsistency argues against a single, shared biological explanation (e.g., ancestry-associated methylation differences at a common set of CpGs) and instead suggests that each clock’s error reflects the specific CpGs and reference populations used during its original training, likely compounded by the underrepresentation of non-White donors in those training cohorts. Regardless of mechanism, these findings indicate that ancestry-related bias is not a marginal concern but a recurring feature of currently available epigenetic clocks, and that reported prediction errors, and any downstream clinical or biological interpretation, should be evaluated separately across ancestry groups rather than assumed to generalize.

The BTECs provided age predictions in normal breast tissue with lower errors than any of the pan-tissue models, including small but significant improvements over the deep-learning-based AltumAge model, without the need for ad hoc, dataset-specific regressions. This performance was assessed using a Leave-One-Cohort-Out (LOCO) approach, ensuring that no data leakage occurred between training and evaluation. BTEC also outperformed existing models on the tumor-adjacent cohort and on independent TCGA samples, both external to the training data, further supporting its generalizability. Unlike AltumAge, BTECs offer greater interpretability, allowing the underlying CpG contributions and associated biological pathways to be examined. The results on breast tissue samples from healthy donors indicate a lack of intrinsic age acceleration, in contrast with prior results reported by Sehl et al. [25].

When applied to tumor-adjacent samples, our models reported significantly lower EAA values compared to the healthy tissue samples. This could indicate epigenetic distortions in the TA samples, either due to the disease or the treatment. Neither the BTECs nor any of the other models tested could validate previous reports of accelerated epigenetic aging in TA samples [22,23]. On the contrary, according to the BTECs results, TA tissue has a tendency toward negative (or decelerated) epigenetic aging.

On breast tumor samples, the BTECs detected a significant decrease in epigenetic aging (EAA < 0). We emphasize that a negative EAA does not imply that tumors are literally ‘biologically younger’ in the sense of delayed aging; rather, it indicates that methylation levels at age-associated CpG sites have shifted in a direction opposite to that expected from normal chronological aging, reflecting a disruption of the epigenetic aging trajectory rather than a reversal of biological age itself. This decrease in EAA comes in contrast with the early observations of accelerated EAA in breast tumors from Hannum [10] and Horvath [9] (later corrected and restricted to HR+ [26]) and the more recent ones from de Lima et al. [28], and the overall positive tumor EAA reported by Castle et al. [29] However, Castle et al. also observed a non-significant negative EAA specifically within the late-stage tumor subgroup of their cohort, a pattern that aligns with our findings and with those of Koka et al., who found no overall EAA difference between normal and tumor samples but reported significant negative EAA specifically in the HER2+ and basal subtypes [24]. This partial overlap between our results and the late-stage subgroup finding in Castle et al., together with our substantially larger tumor cohort (*N* = 1108 vs. ~200) and the platform differences between the two studies (targeted bisulfite sequencing on a custom probe set versus array-based methylation data), may help explain the discrepancy between our overall negative tumor EAA and Castle et al.‘s overall positive finding. Here we show that the BTECs consistently detect a strong trend toward negative EAA values and, moreover, our evaluation of other models in the large integrated dataset of tumor samples (*N* = 1108) suggests that the trend of epigenetic age acceleration reported in some studies cannot be validated. This suggests that the notion of epigenetic age acceleration in breast tumors, taken as an established fact in some studies [22], cannot be sustained. The epigenetic alterations in tumors observed here indicate a decoupling between methylation states and aging which seem to lead to a decrease in epigenetic aging.

Although we found statistically significant associations between BTEC-derived EAA and both fibroblast and immune cell fractions, these associations were modest in magnitude and explained only a small proportion of the overall variance in EAA. Importantly, the molecular subtype-associated EAA differences reported in this study remained robust after accounting for cell-type composition, with subtype explaining substantially more variance than any cell-type covariate. This suggests that the negative EAA we observe, particularly in TNBC tumors, reflects a biological signal associated with tumor subtype rather than a byproduct of differential infiltration by cell populations with distinct baseline methylation age profiles. Nonetheless, we cannot fully exclude the contribution of more granular immune cell subpopulations, and future work using higher-resolution immune deconvolution may help further refine the biological interpretation of subtype-associated epigenetic aging.

We did observe that BTEC-predicted EAA in TNBC samples differed significantly by treatment history, with chemotherapy- or radiation-treated samples showing higher EAA values. This is consistent with prior reports that cytotoxic and radiation-based treatments can alter epigenetic aging [59,60] and suggests that treatment exposure contributes measurably to EAA variance in tumor samples. Treatment history was available for only a subset of samples (TNBC in two datasets), and it should be noted that the public datasets used in this analysis are heterogeneous in origin and metadata completeness. This limited the extent to which we could systematically assess or control for treatment history as a confounder across the broader cohort, and we cannot exclude that undocumented treatment exposure contributes to unexplained EAA variance in the remaining samples.

Notably, when comparing different tumor subtypes, we observed that HER2+ and TNBC tumors exhibited lower EAA than HR+ tumors, in agreement with the study from Koka et al. [24]. This suggests that tumor subtypes influence epigenetic aging patterns in breast tissue. In contrast, we found no significant association between BTEC-derived EAA and tumor grade or stage within the HR+ or TNBC subtypes, suggesting that the subtype-associated EAA differences are not simply a proxy for tumor progression or differentiation status.

BTEC was intentionally trained exclusively on healthy breast tissue, establishing a normal aging baseline against which deviations in tumor and tumor-adjacent samples can be interpreted. Applying a model trained on healthy tissue to tumor samples, which differ substantially in cell composition and microenvironment, raises the question of whether the resulting EAA reflects genuine biological signal or an artifact of extrapolating beyond the training distribution. Several observations support a biological interpretation: BTEC retained strong performance on tumor-adjacent samples (Figure 4), which are external to the training data yet histologically distinct from strictly healthy tissue, outperforming existing clocks in this setting; probe-level analysis revealed a systematic, rather than random, pattern of decorrelation between methylation and age in tumors (Figure 7); the magnitude of EAA varied consistently by molecular subtype in a manner that tracks known differences in tumor aggressiveness (Figure 5); and our findings were broadly concordant with independent reports of negative EAA in aggressive tumor subgroups from Castle et al. [29] and Koka et al. [24] Additionally, the association we observe between BTEC-derived EAA and long-term survival outcomes, while exploratory, provides a further, independent line of evidence that this signal carries biological and clinical relevance beyond a statistical artifact of model extrapolation. Nonetheless, we cannot fully rule out that some portion of the observed deviation reflects model behavior outside its training distribution rather than a purely biological phenomenon, and the altered cell composition and microenvironment of tumor tissue relative to healthy tissue is itself a potential contributor to this signal, one that our cell-type deconvolution analysis suggests only partially explains the observed effect. We therefore interpret BTEC-derived EAA in tumor and tumor-adjacent samples as reflecting a deviation from normal aging patterns, rather than an absolute estimate of biological age, and recommend that future work validate these findings using orthogonal biological measures of tumor aggressiveness and independent cohorts. Candidate biological mechanisms consistent with this altered pattern include epigenetic reprogramming during tumorigenesis, de-differentiation toward stem-like or progenitor-like states, and loss of tissue-specific aging signatures as tumors diverge from normal differentiated breast epithelium; distinguishing among these possibilities will require orthogonal molecular characterization beyond methylation data alone.

The deviation from normal aging patterns captured by negative EAA values observed in breast tumors raises the possibility of targeted therapeutic interventions. If this deviation from normal aging patterns reflects, at least in part, a de-differentiation-like process, this could conceivably suggest treatments aimed at reversing such changes, though this interpretation requires further validation. Anti-aging therapies, such as senescence modulation or telomere uncapping, could hold promise in this context. Senescence is a known response to DNA damage and stress, often acting as a barrier to tumor progression. By targeting the senescent cells within tumors, it may be possible to influence their epigenetic age and potentially improve treatment outcomes. Similarly, telomere uncapping therapies could counteract the epigenetic age distortion seen in tumors, as telomere length and maintenance are closely associated with aging and cellular senescence. Future studies should investigate how such interventions could be combined with traditional treatments to improve efficacy.

The analysis of Ki-67, a widely used proliferation marker [38,39] revealed no consistent relationship between tumor cell proliferation status and the epigenetic age predicted by the BTECs. While two datasets showed that tumors with high Ki-67 expression had significantly lower epigenetic ages and EAAs, consistent with the idea that lower epigenetic age may reflect greater replicative potential, another dataset yielded opposite results. These inconsistencies suggest that the decelerated aging signature detected by BTEC in breast tumors cannot be readily explained by differences in proliferative activity. To further test whether lower predicted epigenetic ages are linked to cell replication history, we applied a mitotic clock (stemTOC [45]) to estimate cumulative cell divisions. Mitotic age showed no significant correlations with chronological age, BTECs’ predictions, or EAA in either tumor or control samples. Together, these findings indicate that the epigenetic age predicted by the BTECs in breast tumors is not simply a proxy for proliferative history. Instead, it may capture other tumor-specific processes that decouple epigenetic age from both cell division dynamics and chronological age. This comes in contrast with a recent study in mice which suggests a clear link between proliferative stage and epigenetic age [61] thus calling for further research into the associations between Ki-67, proliferative state, replication burden and epigenetic and chronological ages in human breast tissue.

Analysis of the genes associated with BTEC probes showed that both models rely on largely overlapping gene sets, but with limited direct connections to established mammalian aging genes.

According to the GenAge database, only a handful of genes with strong links to aging were identified, including SHC1, TP53, HTT, ADCY5, EPS8, GHR, and MTOR, while most mapped genes were related to cancer pathways rather than canonical aging processes. The genes associated with probes positively correlated with age were enriched in protein translation, G protein-mediated signaling, and oncogenic signaling amplification. In contrast, genes linked to negatively correlated probes were enriched in cell adhesion, developmental signaling pathways (especially Wnt, Hedgehog, and ErbB), AMPK signaling, and transcriptional regulation. Notably, both models included oncogenes such as JAK1, TP73, PDGFRA, CCND1, CCND2, WWTR1 (TAZ), and STAT5B, highlighting their relevance to tumor biology. Although both models shared enrichment in G protein signaling among their positive probes, the genes associated with negative probes presented enrichment in divergent terms: the BMIQ-based model was enriched for focal adhesion, whereas the SeSAMe-based model was enriched for Wnt signaling terms.

Stratification of tumors by BTEC-derived EAA revealed distinct transcriptional programs: tumors with EAA < 0 showed overexpression of cell cycle and mitosis regulators (CDC20, CCNA2, PLK1, CDK1) and immune function genes (*IL2RA*), while those with EAA > 0 overexpressed PI3K–Akt signaling components (NTRK2, IGF1R, AREG, FGF1) and prognostic markers such as PGR and SCUBE2. The strong concordance between the two BTEC models supports the robustness of these associations, though the BMIQ-based model identified broader signatures. These findings suggest that BTEC-predicted epigenetic age captures biologically meaningful differences in proliferation, immune activity, and signaling pathways relevant to breast cancer.

Our analysis indicates that the prognostic relevance of BTEC-derived EAA may not be linear but instead could depend on specific thresholds, with cutoffs identifying survival differences in HR+ and TNBC cohorts in our exploratory analysis. These findings suggest that extreme deviations in epigenetic age – rather than the simple distinction between accelerated and decelerated states – may potentially reflect biologically distinct tumor behaviors linked to patient outcomes. Our results, which tentatively suggest that tumors with the most negative EAA values have worse prognosis are in agreement with the observations from Shang et al., which reported worse outcomes for the group of tumors with the lowest EAA in the TCGA [62,63]. Importantly, these exploratory prognostic associations were not captured by established clocks such as Horvath or AltumAge, highlighting the potential added clinical value of BTEC, pending confirmation in independent, adequately powered cohorts. The differences between the BMIQ- and SeSAMe-based models further suggest that distinct CpG sets may capture complementary biological signals, making each model potentially more informative in specific tumor contexts.

Several limitations of this study should be acknowledged. The public datasets used in this analysis are heterogeneous in origin and metadata completeness, which limited our ability to systematically assess certain clinically relevant covariates. For example, BRCA1 mutation status was available in only one dataset with insufficient sample size for meaningful analysis, and obesity status showed no significant association with EAA in either BTEC or other models. Additionally, while we found that sample preservation method (FFPE vs. fresh frozen) did not significantly affect BTEC-derived EAA estimates, this technical variable should be considered in future studies using mixed preservation protocols.

Given that we have demonstrated that the epigenetic age of both healthy and tumor tissue has significant value, an interesting next step would be to explore the integration of epigenetic age with other risk factors, such as breast density. Breast density has long been recognized as an important risk factor for breast cancer, but it currently remains an incomplete predictor of individual risk [64,65]. Combining epigenetic age data with breast density measurements could significantly improve risk models, providing a more accurate picture of a patient’s cancer risk profile. This could prove valuable for both early detection and personalized screening strategies. Further research is needed to determine the best way to incorporate these combined biomarkers into routine clinical practice for risk stratification and monitoring.

In summary, our study shows that breast tissue-specific epigenetic clocks (BTECs) improve age prediction performance compared to pan-tissue models, but their main value lies in the biological and clinical insights they provide. Across a large integrated dataset, neither BTECs nor other established clocks detected consistent evidence of accelerated aging in breast tumors; instead, tumors displayed a robust trend toward negative epigenetic age accelerations. This suggests a mechanistic decoupling of methylation aging signals from both chronological age and proliferative history, potentially reflecting tumor-specific processes such as de-differentiation, stemness, or epigenetic reprogramming. Beyond prediction accuracy, BTECs revealed distinct transcriptional programs and subtype-specific differences and identified prognostic patterns in HR+ and TNBC tumors that were not captured by earlier clocks. These findings highlight the broader significance of tissue-specific epigenetic clocks: not only as biomarkers for refining risk stratification, but also as tools for uncovering the biological underpinnings of cancer-associated aging distortions.

## Methods

### Study cohorts

We used publicly available methylation data from female subjects from 13 previous studies, which we retrieved from the GEO database [66,67]. We include the GEO accession number, the number of samples per condition and tumor subtype (when available), the minimum, maximum, and median ages of the subjects, the type of data provided (either raw IDAT files, beta values or M and U values), the type of methylation array used and the corresponding references in Supplementary Table 1. The data includes methylation data from healthy breast tissue samples, tumor-adjacent samples (histologically normal breast tissue, adjacent to a tumor site) and breast tumor samples.

### Data collection and preprocessing

Methylation data from previous studies were obtained from the GEO database. Whenever raw data was provided, we processed in parallel with the SeSAMe [34] and Minfi [32] R packages to obtain beta values. SeSAMe was run through the ‘openSesame’ wrapper with the QCDPB option for pre-processing (probe masking, channel inference, dye bias correction, *P*-value masking using oob and background subtraction using oob). In the case of Minfi, beta values were obtained using the getBeta function with standard parameters and an offset value of 100. The beta values produced by Minfi were then BMIQ-normalized using the wateRmelon [33] R package.

When only beta values were available (Supplementary Table S1), we added them as provided to the ‘SeSAMe-normalized’ data and we applied BMIQ-normalization via wateRmelon before adding them to the ‘BMIQ-normalized’ data.

In the cases in which only methylated and unmethylated signals and *P*-values were provided (M, U and P), we masked those values with *P* > 0.01 and then calculated the beta values using the formula:

$$\beta =\frac{M}{M+U+100}$$

where M and U are the intensities of the methylated and unmethylated signals, respectively.

We modified the original sample labels as follows: we labeled as ‘Tumor adjacent’ all samples with the annotations ‘adjacent normal’ and ‘ipsilateral normal,’ samples with ‘reduction mammoplasty’ and ‘prophylactic’ were relabeled as ‘Healthy donor,’ and samples annotated as ‘DCIS’ were labeled ‘Tumor.’ Samples labeled ‘controlateral’ were discarded.

The beta values from the different datasets were merged by cohort (Healthy, Tumor adjacent, and Tumor) using an outer join, so that we preserved CpG probes even if they were only present (or not masked during the quality control) in a few datasets. Missing values were imputed using *k*-nearest neighbors via pyaging (see below).

### Tumor purity estimation

We employed the InfiniumPurify R package to obtain tumor purity estimations on every sample in our integrated dataset, setting the ‘tumor.type’ option to BRCA in all cases.

### Epigenetic age prediction using existing models

We used four pan-tissue epigenetic clocks to predict chronological age using DNA methylation data from breast tissue of healthy donors using the PyAging Python library [68]: Horvath[9] Hannum[10]PhenoAge [21] and AltumAge [28]. We imputed missing beta values using the *k*-nearest neighbors strategy separately on the normal, tumor-adjacent and tumor samples, with *k* = 5. The proportion of imputed CpGs per sample was generally low across clocks, platforms (450K, EPIC) and cohorts (median 0.3–14.1%; Supplementary Table S24), with a small subset of samples showing higher missingness. To assess whether imputation affected prediction accuracy, we tested the correlation between EAA/prediction error and the percentage of imputed CpGs on the three cohorts (Normal, Tumor adjacent, Tumor) for each clock and dataset. Effect sizes on the larger datasets (*N* > 100) were consistently negligible (*r*^2^ < 0.036) and only reached higher values (*r*^2^ > 0.2) in tests with less than 20 samples. None of the correlations tested were found to be statistically significant (FDR < 0.05, *n* = 122 tests; Supplementary Table 25, Supplementary Figure 10), indicating that missingness and its imputation did not meaningfully bias epigenetic age predictions.

### Residuals-based EAA predictions analysis

To evaluate whether the linear relationship between chronological age and predicted age (used to compute residual-based EAA) is stable across reference datasets (healthy donor samples), we fitted an ordinary least squares model of the form Age ~ PredictedAge * Dataset for each of the four epigenetic clocks (AltumAge, PhenoAge, Hannum’s, Horvath’s), using the ols function from the statsmodels.formula.api Python package. This model allows both the intercept and slope of the regression to vary by reference dataset. We then performed a Type II ANOVA (anova_lm, statsmodels.stats.anova) on each fitted model to test the significance of the Dataset main effect (differences in intercept across datasets) and the Predicted Age: Dataset interaction term (differences in slope across datasets). We additionally computed residual-based EAA for each clock and normalization method using linear models fitted separately for each reference dataset, and compared the resulting prediction error (RMSE and MAE) to the error obtained using raw EAA (predicted age minus chronological age, without residualization).

### Mitotic age predictions

We used the recent stemTOC^45^ model to obtain estimates of the mitotic age of every sample in the integrated dataset. Following the details in the original publication, we ‘define the mitotic age of the sample as the 95% upper quantile of DNAm values over the 371 vivo-mitotic CpGs.’

### BTEC training

We used elastic net to train chronological age predictors based on the beta values of cross-platform (450k, EPIC, EPICv2) probes which exhibited high correlation with age in the set of healthy samples. Specifically, we considered the top 5% probes ranked by the absolute Pearson correlation between chronological age and either the probe β values or their squared values (β^2^), under both normalization methods (|*r*| >0.321, *N* = 7048 for BMIQ and |*r*| > 0.280, *N* = 7460 for SeSAMe). This resulted in a total of 4713 probes common to both approaches (Supplementary Table 3).

An Elastic Net model development was performed using 553 samples from healthy subjects across seven datasets. We set the mixing parameter (L1 ratio) to 0.5 and then optimized the regularization strength (alpha) values to 0.0077 through 5-fold cross-validation on both the BMIQ- and the SeSAMe-normalized data.

To obtain an unbiased estimate of the model’s generalizability across cohorts, we applied a leave-one-cohort-out (LOCO) validation strategy. On each of the seven iterations, one dataset was held out as an independent test set, while the model was trained on the remaining six datasets using the fixed hyperparameters determined earlier. Because hyperparameter selection was completed before cohort-level validation and no information from the held-out cohort was used during model fitting within each iteration, this procedure provides an unbiased assessment of performance on independent datasets and avoids data leakage. Furthermore, evaluating every cohort as the test set demonstrates that model performance is not dependent on an arbitrary choice of validation cohort.

After the LOCO-based performance evaluation, the final models were retrained using the 553 healthy samples to maximize the amount of training data available. These final BTECs were used for age predictions on the tumor adjacent, TCGA’s and tumor samples.

### BTEC’s association with cell-type composition

To assess whether tumor-associated EAA differences could be confounded by cell-type composition, we estimated epithelial (Epi), fibroblast (Fib), and immune cell (IC) fractions for each tumor sample using the R implementation of EpiDISH [37]. We then tested the association between each cell-type fraction and BTEC-derived EAA using Pearson correlation, and fitted a linear model of EAA as a function of Epi and Fib fractions (IC omitted as fractions sum to 1) using ordinary least squares regression. To assess whether molecular subtype-associated EAA differences persisted after accounting for cell composition, we additionally fitted an ANCOVA-type model including molecular subtype together with Epi and Fib fractions as covariates, and evaluated significance using a Type II ANOVA. All analyses were performed separately on BMIQ- and SeSAMe-normalized data.

### Validation on TCGA data

We collected breast tissue DNA methylation data from a total of 97 samples from the breast cancer cohort of the Cancer Genome Atlas (TCGA) [36] with available raw methylation data and chronological age values. Of these, we discarded 53 which were present in the training set of AltumAge. The remaining 44 samples were processed like the samples from the integrated dataset and used for chronological age prediction with the different models.

### Transcriptomic data analysis

We used transcriptomic data from two datasets to 1) compare the transcriptomic profiles of tumors with EAA > 0 and EAA < 0 and 2) analyze the correlations between gene expression and age.

To compare tumors with in different EAA groups, we used the dataset GSE37754 from Terunuma’s et al. study [56] which contained microarray expression data (Affymetrix Human Gene 1.0 ST Array) from 108 tumor samples, out of which 55 matched tumor samples from the same study with available methylation data (GSE37751). The data were mean-centered and scaled to unit variance and then the two groups were compared using a two-sided Wilcoxon test.

To study the correlation between gene expression and aging in breast tissue, we used the transcriptomic data from Song’s et al. study [47] (GSE102088), which included 104 samples from healthy donors. We used the normalized gene expression matrix provided by the authors to determine the Pearson’s correlation coefficient of each gene and chronological age.

### Gene set enrichment analysis

In all cases, we used the GSEAPY Python library [69] to perform gene set enrichment analysis through the Enrichr [70] API. We included the KEGG 2021, GO Molecular Functions 2025, GO Biological Process 2025 and WikiPathways 2024 gene sets to perform the analysis on human genes, with no specific background and an adjusted *P*-value cutoff of 0.2.

To conduct enrichment analysis on genes associated to sets of CpG probes, the probes were mapped to genes using the Illumina array manifest annotations. For CpG probes annotated to multiple genes, all annotated genes were retained. CpG probes without an annotated gene symbol were excluded. Because multiple CpG probes can map to the same gene, duplicate gene symbols were collapsed to a unique gene list prior to enrichment analysis, such that each gene was represented only once.

### Survival analysis

The hazard ratio estimations were obtained using a Cox proportional hazard model through the lifelines Python library [71]. Kaplan–Meier survival curves were obtained using the same library.

To explore potential non-linear associations between epigenetic age acceleration (EAA) and clinical outcomes, we performed an exploratory cutoff-based survival analysis. For each EAA model (BMIQ- and SeSAMe-based), patients were stratified into two groups across a range of potential cutoff values. At each cutoff, a log-rank test was computed to compare survival distributions between the two resulting groups. To ensure sufficient statistical power and group balance, only cutoffs that produced groups each containing at least 20% of the total cohort were considered.

### Bootstrapping for cutoff validation

To assess the stability of the identified optimal cutoff values, we performed a bootstrap validation procedure. For each cohort and EAA model, bootstrapped samples were generated by drawing *2N* observations with replacement from the original dataset, where *N* is the number of subjects in the cohort. This procedure was repeated 100 times. Within each bootstrap iteration, the optimal cutoff was recalculated, and the corresponding log-rank *P*-value was recorded. The distribution of optimal cutoffs across bootstrap replicates was summarized using the median and 95% confidence interval. A cutoff was considered validated if it fell within the 95% confidence interval and if it yielded a *P*-value < 0.05 in at least 95 of the bootstrap samples.

### Statistical analysis

Unless otherwise specified, all statistical comparisons between groups were performed using a two-sided Mann-Whitney U test, with a significance threshold of α = 0.05 (95% confidence level). To account for multiple hypothesis testing, *P*-values were corrected using the Benjamini–Hochberg false discovery rate (FDR) procedure, with a significance threshold of FDR < 0.05.

## Supplementary Material

Supplementary_Figures_clean.docx

## Data Availability

All the data used in this study is publicly available. Data sources are detailed in Supplementary Table 1. To increase reproducibility and facilitate further studies, the normalized data has been uploaded to figshare, with http://doi.org/10.6084/m9.figshare.30489026 and will be released upon publication.

## Code availability

This study did not generate any new computational methods. The specific Python libraries used are detailed in the Methods section above.

The Python code used to train the BTEC models is provided at www.github.com/leo-gg/BTEC.

Any additional information required to reanalyze the data reported in this paper is available from the lead contact upon request: Miguel Quintela-Fandino (m.quintela@cnio.es).
