# Supplementary material for: A breast tissue-specific epigenetic clock provides accurate chronological age predictions and reveals de-correlation of age and DNA methylation in tumor-adjacent and tumor samples
Source: Epigenetics. 2026 Sep 11;21(1):2714582. doi: 10.1080/15592294.2026.2714582 (PMC13577255; doi:10.1080/15592294.2026.2714582)
Supplement: Supplementary file 1 [file KEPI_A_2714582_SM7159.docx]

**Supplementary Figure 1.** Distribution of DNA methylation-based estimated tumor purity on the normal breast tissue (N=553), tumor adjacent (TA, N=362) and tumor samples (N=1108). Boxes represent the quartiles of the distributions, and whiskers extend to 1.5 times the inter-quartile range. Points beyond these values are considered outliers and drawn individually. The asterisks indicate significant differences (p<0.05).

**Supplementary Figure 2.** Instability of the residual-based EAA correction across reference datasets. Each panel shows linear fits of chronological age (y-axis) against raw predicted epigenetic age (x-axis), with each colored line representing the fit obtained using a different healthy breast tissue dataset as the reference cohort (see legend). Rows correspond to clocks (AltumAge, PhenoAge, Hannum, Horvath); columns to normalization method (BMIQ, SeSAMe). The marked divergence in slope and intercept across references shows that residual-based EAA correction is highly dependent on the reference dataset used, consistent with the Type II ANOVA results in Supplementary Table 4.

**Supplementary Figure 3.** Distribution of prediction error after residual-based EAA correction using different reference datasets. RMSE (top) and median absolute error (MAE, bottom) were computed for each epigenetic clock and normalization method (BMIQ, SeSAMe) after residualizing predicted age on chronological age using each of several healthy-donor reference datasets in turn (points, one per reference dataset); box plots summarize the resulting distribution across references. Red diamonds indicate the baseline error obtained from raw (non-residualized) predictions. Naïve (constant-age prediction) and Random baselines are shown for comparison.

**Supplementary Figure 4.** Chronological ages of the normal breast, tumor adjacent and tumor samples and the corresponding ages predicted by Horvath’s model and AltumAge based on BMIQ- and SeSAMe-normalized data.

**Supplementary Figure 5.** Distributions of epigenetic age acceleration values assigned by BTEC to samples from the GSE2254845 (A) and GSE72308 (B) datasets, stratified by tumor molecular subtype. Asterisks indicate significant differences (p<0.05, one-sided Wilcoxon test).

**Supplementary Figure 6.** **A**. Distribution of predicted epigenetic age by Ki-67 group on three different datasets (SeSAMe-normalized data). **B.** Distribution of EAA by Ki-67 group on the same datasets. **C.** stemTOC’s predicted mitotic age plotted against chronological age (left), BTEC’s epigenetic age (middle) and BTEC’s EAA on all the tumor samples of the integrated dataset (N=1108, SeSAMe normalization).

**Supplementary Figure 7.** **A**. stemTOC’s predicted mitotic age plotted against chronological age (left), BTEC’s epigenetic age (middle) and BTEC’s EAA on all the normal breast and tumor-adjacent samples of the integrated dataset (BMIQ normalization). **B**. Same as A using the SeSAMe-normalized data. **C**. Predicted mitotic ages and predicted age (top) and EAA (bottom) by Horvath’s clock and AltumAge using BMIQ- (left) and SeSAMe-normalized (right) data.

**Supplementary Figure 8.** Functional (KEGG, WikiPathways, GO Molecular Function) terms enriched in the set of genes associated to probes with positive coefficients in the BMIQ-based BTEC (**A),** probes with negative coefficients in the BMIQ-based BTEC (**B**), probes with positive coefficients in the SeSAMe-based BTEC (**C**) and probes with negative coefficients in the SeSAMe-based BTEC (**D**).

**Supplementary Figure 9.** Log-rank test statistic value obtained using different Horvath’s EAA cutoff values to split patients from the HR+ survival dataset (GSE37754) into two groups and comparing their survival times (left). None of the possible cutoffs resulted in a could be used to generate groups with significant differences in the HR+ survival data. Bootstrapping results on the same subjects, showing the distribution of optimized EAA values and the corresponding p-values across the 100 bootstrapped samples (middle and right). The green arrows indicate the value of the optimal cutoff found in the original data. The dashed red line marks the significance thresholds for the log-rank test statistic and the p-value. The confidence interval of the optimal EAA cutoff found across the 100 bootstrapped samples is highlighted in pink.

**Supplementary Figure 10**. Relationship between the percentage of imputed CpG probes and epigenetic age acceleration (EAA) across clocks and normalization methods. Each panel shows per-sample EAA (predicted minus chronological age) plotted against the percentage of imputed CpG probes for one clock (AltumAge, Horvath, Hannum, PhenoAge) and normalization method (SeSAMe, BMIQ), colored by cohort (Normal, TA, Tumor). No consistent relationship between missingness and EAA magnitude or direction is apparent across clocks, cohorts, or normalization methods.
